# The Specification and Functional Maturation of Sub-Cerebral Projection Neurons Derived from Human Induced Pluripotent Stem Cells

**DOI:** 10.1177/15473287251399623

**Published:** 2025-12

**Authors:** Zachary Jordan, Anukriti Singh, Seth Kattapong-Graber, Sonal Goswami, Neville E. Sanjana, Emanuela Zuccaro, Feng Zhang, Kevin Eggan, Jenny Hsieh, Brian P. Hermann, Jean M. Hebert, Asif Mirza Maroof

**Affiliations:** 1 Department of Neuroscience, Developmental and Regenerative Biology, The University of Texas at San Antonio, San Antonio, Texas, USA.; 2 Brain Health Consortium, The University of Texas at San Antonio, San Antonio, Texas, USA.; 3 Department of Neuroscience, Albert Einstein College of Medicine, Bronx, New York, USA.; 4 New York Genome Center, New York, New York, USA.; 5 Department of Biology, New York University, New York, New York, USA.; 6 Department of Stem Cell and Regenerative Biology, Harvard University, Cambridge, Massachusetts, USA.; 7 Broad Institute of MIT and Harvard, Cambridge, Massachusetts, USA.; 8 McGovern Institute for Brain Research at MIT, Cambridge, Massachusetts, USA.; 9 Department of Brain and Cognitive Science, Massachusetts Institute of Technology, Cambridge, Massachusetts, USA.; 10 Department of Biological Engineering, Massachusetts Institute of Technology, Cambridge, Massachusetts, USA.; 11 Howard Hughes Medical Institute, Chevy Chase, Maryland, USA.

**Keywords:** sub-cerebral projection neurons, FEZF2-GFP reporter, fate specification

## Abstract

Human induced pluripotent stem cells (hiPSC) are an invaluable resource for investigating the molecular mechanisms regulating cell fate specification during brain development. However, most directed differentiation methods exhibit significant cell fate heterogeneity and require several months to become functional. To address this challenge, we developed a green fluorescent protein (GFP) reporter system in hiPSC by targeting the genomic locus of Forebrain Enriched Zinc Finger 2 (*FEZF2*), which encodes a transcription factor essential for the fate specification of sub-cerebral projection neurons (SCPN) during forebrain development. Using this FEZF2-GFP reporter hiPSC line, we optimized a directed differentiation protocol to rapidly and efficiently generate pallial progenitors and glutamatergic neuronal subgroups after 3 weeks. Through fluorescence activated cell sorting for both GFP and CD200, isolated post-mitotic SCPN immediately displayed electrophysiological properties and formed glutamatergic synapses within 4 additional weeks of *in vitro* cell culture. Co-culture with hiPSC-derived spinal motor neurons further enhanced these electrophysiological characteristics, improved viability, and increased synapse formation in SCPN. This study presents a streamlined and effective strategy to generate, isolate, and characterize human motor neuron circuits, providing insights into the molecular determinants regulating synaptogenesis and functional maturation.

## Introduction

The cerebral cortex emerges from an initially pseudostratified sheet of neuroepithelial cells (NEC) in the dorsal telencephalon (i.e., pallium) that gives rise to radial glia cells (RGC).^1^ RGC generate neurons through direct neurogenesis and indirectly through transit-amplifying intermediate progenitor cells (IPC).^2^ Important questions remain regarding the signals that promote the transition from NEC to RGC subtypes, and whether those RGC subtypes correspond to the fate specification of glutamatergic projection neuronal subgroups.

Single-cell RNA sequencing (scRNA-seq) of human fetal tissue has provided key insights about the cellular diversity and signaling networks involved in cortical development, particularly during the stages of peak neurogenesis.^3-6^ However, due to the scarcity of human fetal tissue, patterning human induced pluripotent stem cells (hiPSC) using *in vitro* approaches has become the most viable solution for capturing the complexity and unique features of cell fate specification during human cortex development.^7-9^

Forebrain Enriched Zinc Finger 2 (*FEZF2*) encodes a transcription factor essential for the fate specification of sub-cerebral projection neurons (SCPN). FEZF2 induces the expression of genes required for axonal projections to sub-cerebral targets while maintaining an excitatory glutamatergic neurotransmitter profile.^10^ Studies in mice found that FEZF2 regulates the fate specification of RGC into SCPN by selectively repressing the expression of genes associated with alternative projection neuronal subtypes.^11^

Given FEZF2’s critical role in temporal fate selection within the developing mouse cortex and previous work on its spatiotemporal expression during human cortex development,^10,12^ we sought to provide an efficient and scalable cell-based platform for studying early human cortical development with hiPSC, enabling the detailed investigation of glutamatergic neuronal fate specification, functionality, and synaptic integration *in vitro*.

## Methods

We used a transcription activator-like effector nuclease (TALEN)-based gene editing approach to target the *FEZF2* locus in a healthy control hiPSC line, enabling the insertion of an enhanced green fluorescent protein (GFP) cassette inframe with the *FEZF2* coding sequence in order to preserve the bi-allelic expression of *FEZF2* together with GFP.^13^ The resulting hemizygous *FEZF2*-GFP line, 18a12, underwent novel monolayer-based directed differentiation to enrich for pallial lineages after 3 weeks. Cultures then underwent fluorescence activated cell sorting (FACS), gated for a combination of the fluorescence of the GFP protein, anti-CD200-APC, and anti-CD44-PE. Directed differentiation conditions were optimized by the addition of three small-molecule inhibitors, SU5402, GDC0623, and YO-01027 (SUGY), to maximize the number of FEZF2-GFP+/CD200+ cells. scRNA-seq from the reporter and the parental hiPSC lines were integrated with human fetal cortical data from a published atlas using Harmony,^14^ visualized by uniform manifold approximation and projection (UMAP) clustering with Seurat, and annotated based on cell type-specific marker expression. The reporter line scRNA-seq data were then visualized using unbiased t-distributed stochastic neighbor embedding (tSNE) clustering to analyze with Velocyto. Single-cell transcriptomes of *FEZF2*-expressing cells were further grouped by the hierarchical clustering of differentially expressed genes (DEG) and compared using gene ontology (GO) to annotate the gene products. Sorted and unsorted populations were assayed by quantitative real-time polymerase chain reaction (qRT-PCR) for numerous cell type-specific neuronal markers and in parallel, replated overnight and immunostained for neuronal proteins or 5-ethynyl-2’-deoxyuridine (EdU) to mark cell division after sorting (Supplementary Table S1). Sorted FEZF2-GFP+/CD200+/CD44− cells (putative SCPN) were cultured on multi-electrode array (MEA) plates for periodic extracellular spike recordings throughout 31 days post-replating (DPR). Parallel cultures of 18a12 hiPSC underwent directed differentiations toward spinal motor neurons (SMN) or cortical GABAergic interneurons (CGIN), each sorted for CD200+/CD44−. Monoculture or co-culture combinations of putative SCPN, SMN, and CGIN were then replated at a uniform 50,000-cell-total onto coverslips for patch-clamp electrophysiological evaluation as well as the immunofluorescence quantification of surviving cells and synaptic puncta. For additional information, see Supplemental Methods.

## Results

### TALEN-mediated genomic targeting to generate FEZF2-GFP hiPSC lines

**FIG. 1.**
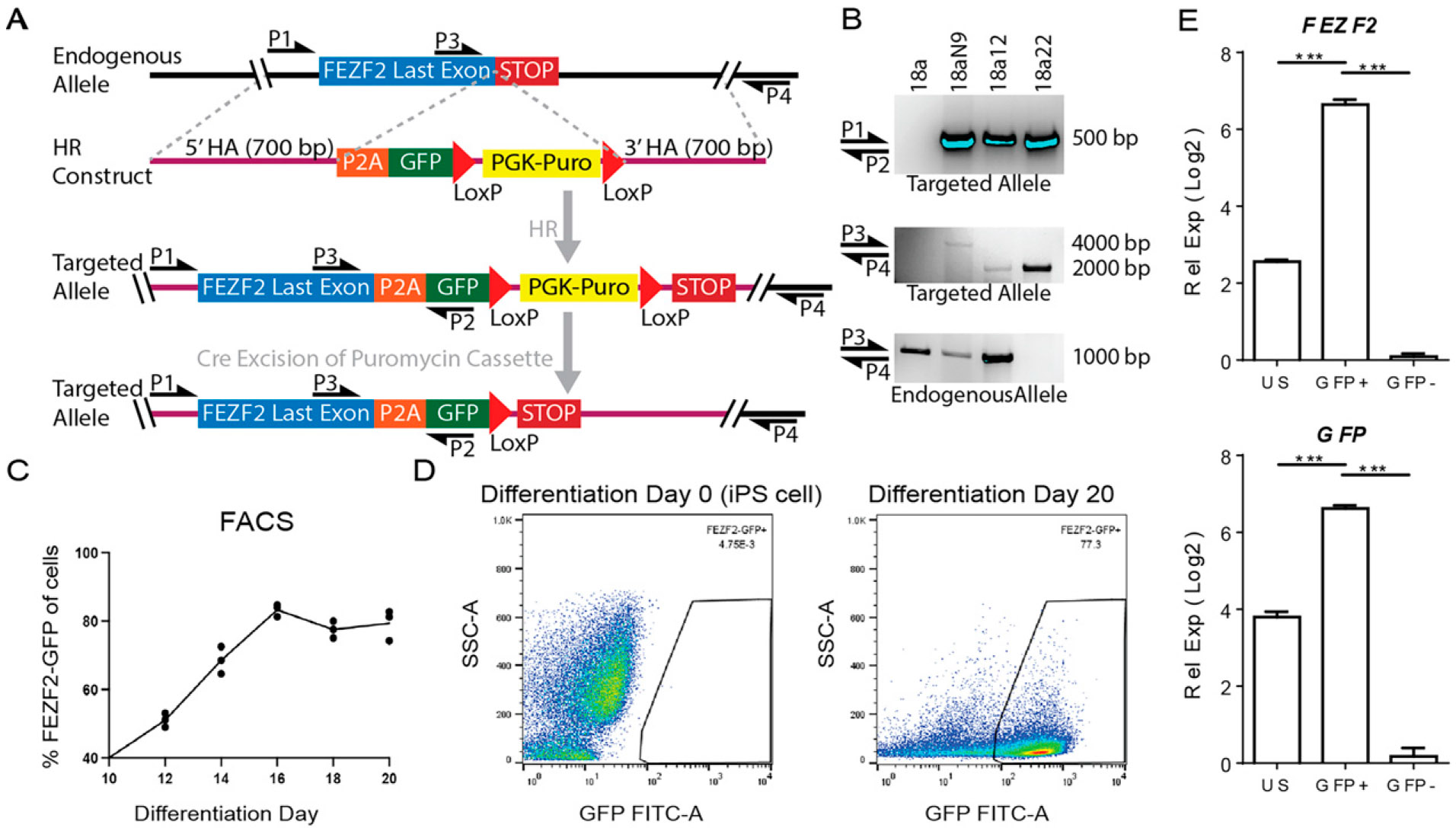
Stable genomic integration upon targeting the *FEZF2* locus of human induced pluripotent stem cells (hiPSC). (**A**) Transcription activator-like effector nuclease (TALEN)-based strategy to target the *FEZF2* genomic locus with enhanced green fluorescent protein (GFP). The homologous recombination (HR) construct contained the homology arm (HA) sequences of 700-base pairs (bp) both upstream and downstream of the *FEZF2* stop codon, which surrounded the P2A-EGFP-LoxP-PGK-Puro-LoxP transgenic sequence. Transfection with the HR construct and plasmids encoding a custom-designed pair of TALEN proteins enabled a targeted, double-stranded cut in the 3′ region of the *FEZF2* locus immediately upstream of the stop codon in hiPSC. Transfected hiPSC colonies that survived puromycin (Puro) selection were then transiently transfected with Cre recombinase to excise the Puro cassette. (**B**) The hiPSC line 18a was transfected with the HR construct and TALEN pairs, and the Puro-resistant clone was designated 18aN9. The 500-bp PCR product from P1 to P2 validated 5′ integration within the last exon of *FEZF2* with P2A-EGFP (*top panel*). The size of the PCR product from P3 to P4 determined whether 3′ integration occurred. The 4000-bp PCR product spanning the full-length insertion past the 3′ HA was detected only in 18aN9. Puro cassette excision was validated by the detection of a 2000-bp PCR product in both 18a12 and 18a22 hiPSC lines (*middle panel*). The 1000-bp band confirmed the presence of the endogenous allele in 18a, 18aN9, and 18a12 (*bottom panel*). (**C**) The percentage of FEZF2-EGFP+ cells of all live cells quantified using fluorescence activated cell sorting (FACS) over 2-day intervals. Each value (dot) and average (line) from *N* = 3 independent differentiations. (**D**) FACS measurements represented as dot plots of GFP fluorescence intensity (GFP FITC–A) vs. side scatter area (SSC–A) of 18a12 hiPSC before (day 0) and after 3 weeks (day 20) of directed differentiation. (**E**) Transcript measurements using quantitative real-time polymerase chain reaction PCR (qRT-PCR) with relative expression (ΔΔCT) of *FEZF2* (*top*) and EGFP (*bottom*) calculated using cycle threshold (CT) values of the given gene after subtracting from glyceraldehyde-3-phosphate dehydrogenase (*GAPDH*), and then normalized within samples to the lowest expressing sample (Log2) mean ± standard deviation (SD) for *N* = 3 differentiations. Analysis of variance with Dunnett’s multiple comparisons test ***P < 0.001. hiPSC, human induced pluripotent stem cells.

To generate stably integrated FEZF2-GFP hiPSC lines, we designed TALEN pairs targeting the genomic region immediately upstream of the stop codon of the human *FEZF2* gene (Fig. 1A).^11^ The 18a hiPSC line derived from a healthy female individual^15^ was transfected with TALEN pairs and the homology-directed repair (HR) construct bearing the *FEZF2*-P2A-EGFP sequence. PCR confirmed the stable integration of the transgene (P1 to P2) and the targeted region, spanning downstream of the 3′ homology arm (P3 to P4) (Fig. 1A, B). Cre recombinase excision of the floxed puromycin cassette was validated by PCR in two clones, revealing the genomic targeting of one allele (18a12; *FEZF2^GFP/+^*) or both alleles (18a22; *FEZF2^GFP/GFP^*) (Fig. 1B). The hiPSC line with the one allele targeted was selected for subsequent experiments. Upon the directed differentiation of the *FEZF2^GFP/+^* hiPSC line toward the pallium,^16^ native GFP expression was first detectable after 8 days (data not shown), with fluorescence intensity plateauing by 16 days (Supplementary Fig. S1A), which was quantified using FACS at 2-day intervals (Fig. 1C, D). Because an effective antibody to FEZF2 was not commercially available, qRT-PCR analysis confirmed significant enrichment of both *FEZF2* and *GFP* transcripts in FEZF2-GFP+ sorted cells (Fig. 1E). Previous work shows that FEZF2 represses the expression of callosal projection neuron genes by repressing *SATB2*^17^ while enhancing the expression of SCPN genes,^10^ which is consistent with our results (Supplementary Fig. S1B). Therefore, GFP expression accurately reports on *FEZF2* expression, with most differentiated cells specified to pallial fates after 3 weeks.

#### A small-molecule cocktail enhances post-mitotic SCPN specification.

Given that *FEZF2* is expressed in both pallial progenitors and glutamatergic neurons,^18^ we sought to enhance neuronal specification while also limiting the pool of cycling progenitors. While standard protocols for patterning hiPSC into cortical projection neurons typically require up to 4 months,^19-23^ we identified an accelerated approach using three small-molecule inhibitors (i.e., SUGY) targeting fibroblast growth factor receptor (FGFR), mitogen-activated protein kinase kinase, and *γ*-secretase signaling pathways, which together regulate NEC, RGC, and IPC self-renewal and cell division.^24^ SUGY treatment significantly enhanced neuronal specification, with no significant differences in GFP expression by day 20 (Supplementary Fig. S2A-E).

#### FEZF2 *expression is enriched in pallial progenitors and glutamatergic neurons*.

**FIG. 2.**
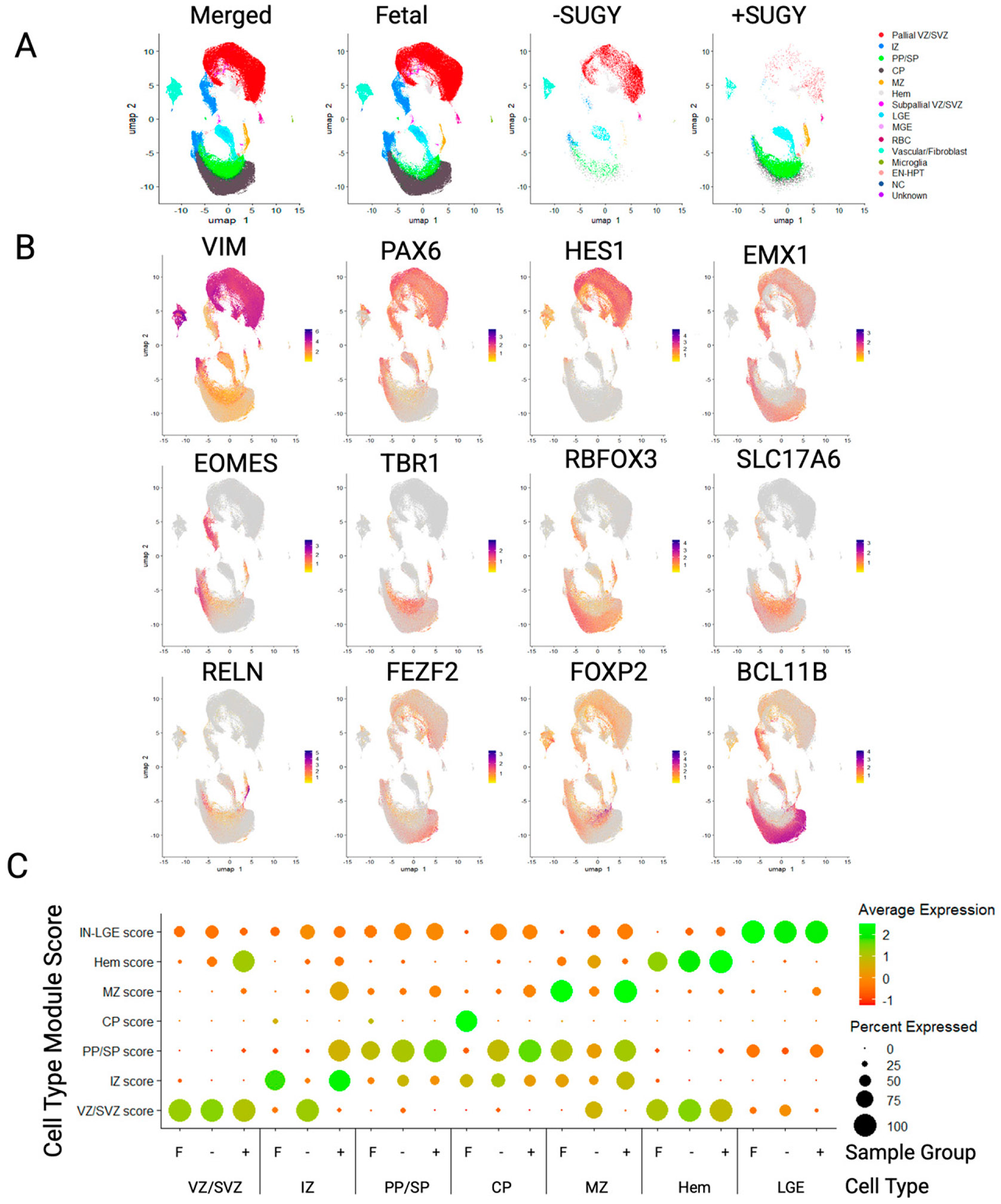
FEZF2-EGFP and parental hiPSC line share common cell type clusters with human fetal forebrain and cortical tissue cells. Single-cell RNA sequencing libraries were made from 18a12 and 18a hiPSC differentiated to day 21 with (+) or without (−) SUGY treatment. (**A**) Harmony^14^ integration with fetal cortical samples from 7 to 16 gestational weeks (GW) from a developmental fetal brain atlas^25^ showing the sample groups and integrated cell type clusters. (**B**) Feature plots showing integrated marker gene expression from hiPSC-derived cells. (**C**) Dot plots showing the marker module score consistency between fetal and hiPSC-derived cells in each region-associated cluster (*y*-axis) for both fetal (F) and hiPSC-derived cells without (−) or with (+) SUGY treatment (*x*-axis). VZ/SVZ, ventricular zone/subventricular zone; IZ, intermediate zone; PP/SP, preplate/subplate; CP, cortical plate; MZ, marginal zone; LGE, lateral ganglionic eminence; MGE, medial ganglionic eminence; RBC, red blood cell; HPT, hypothalamus; NC, neural crest.

To determine the extent to which pallial differentiated hiPSC resembled gene expression signatures associated with human forebrain development, scRNA-seq was performed on the unsorted populations of the *FEZF2^EGFP/+^* hiPSC line and the parental 18a line after 3 weeks of differentiation with or without SUGY treatment. These cells were integrated with the first trimester and early second trimester fetal cortical and forebrain scRNA-seq data from a published atlas.^25^ Data were integrated using Harmony^11^ and visualized using UMAP with Seurat v4^26^ (Fig. 2A). The integrated clusters were annotated based on cell type-specific marker expression (Fig. 2B, Supplementary Fig. S3). The clusters included many early-born cell types, including glutamatergic neuronal subgroups from the marginal zone (MZ), cortical plate (CP), subplate (SP), and preplate (PP) neurons. SP neurons were molecularly indistinguishable from PP neurons at these stages and were therefore grouped into the same cluster. Additionally, in the younger samples and hiPSC-derived datasets, there was a cortical hem NEC cluster with hem-specific markers (*LIM homeobox transcription factor 1 alpha, r-spondin 2*), which is consistent with the hem being a predominant source of Cajal–Retzius (CR) neurons.^27-29^ The clustering validated with dot plots showed canonical cell type-markers in modules between the fetal and hiPSC-derived data (Fig. 2C).

To determine what developmental stage our hiPSC-derived cells most resemble at 3 weeks, we took the 2,000 most variable genes in the combined fetal and hiPSC-derived datasets and compared the Pearson correlation coefficients of the log-transformed counts between the hiPSC-derived data and the fetal data from different age ranges (Supplementary Fig. S4A). The correlations were run between the groups containing multiple samples across age ranges rather than at specific ages to ensure multiple biological replicates and the sufficient representation of all cell types for comparison. Three-week cultures derived from hiPSC with or without SUGY treatment had the highest correlation with the fetal samples from gestational week (GW) 7–9 compared with the samples at GW10–16 (Supplementary Fig. S4A), consistent with SUGY treatment enhancing the fate commitment of progenitors rather than accelerating maturation. The SUGY-treated cells had mostly neurons compared with the nontreated cells, which were mostly progenitors (Supplementary Fig. S4B).

**FIG. 3.**
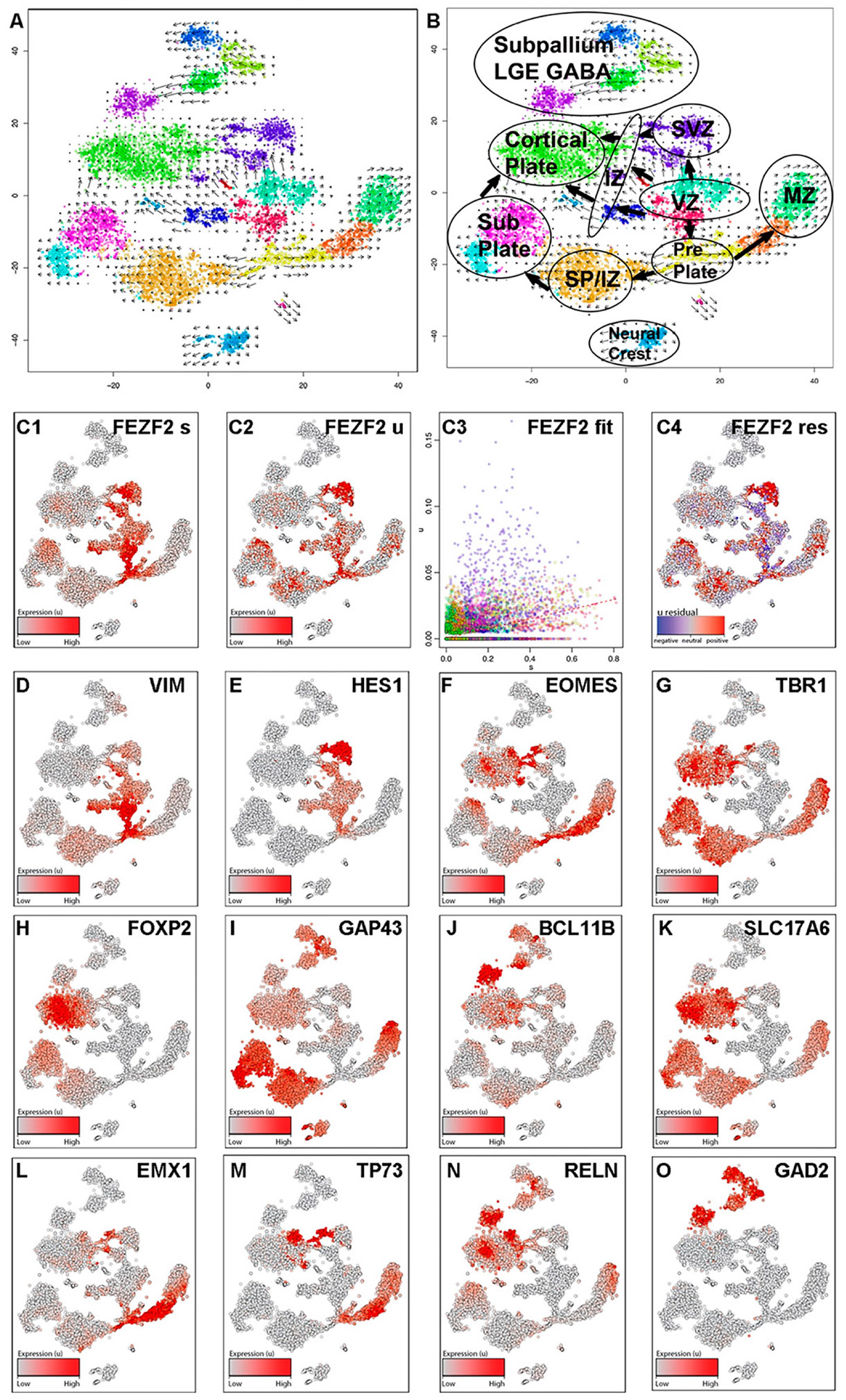
*FEZF2* expression occurs in pallial progenitors that give rise to distinct domains of glutamatergic projection neuron subgroups associated with early fetal development. Single-cell RNA sequencing libraries were made from 18a12 hiPSC differentiated to day 21 with SUGY treatment from day 10 to day 14. (**A**) Velocity analysis of t-distributed stochastic neighbor embedding (tSNE) plot from two independent differentiations. The observed state (dots) and extrapolated future state (vector field overlay) are shown on the tSNE plot. (**A–B**) tSNE plot annotated with subpopulations associated with fetal forebrain regions and their cell fate specification trajectories overlayed. Subpopulations were inferred based on cell type-specific markers in Figures 3 and 4 and Supplementary Figure S5. (**C1–C2**) Feature plots of the transcript expression pattern of *FEZF2* spliced (**C1**) and unspliced **(C2). (C3)** A phase portrait of *FEZF2* unspliced (u, *y*-axis) vs. spliced (s, *x*-axis) transcripts. The *γ* fit line in *red* is determined by the extreme quantile fit procedure and shows the expected steady state of nascent to mature transcripts, above which *FEZF2* transcription is upregulated **(C3). (C4)** Residual plot (unspliced—spliced) *FEZF2* transcript expression that shows comparatively higher nascent transcripts in red and comparatively higher mature transcripts in *blue*. (**D–O**) Feature plots illustrating the spliced transcript abundance patterns of *VIM* (**D**), *HES1* (**E**), *EOMES* (*TBR2*; **F**), *TBR1* (**G**), *FOXP2* (**H**), *GAP43* (**I**), *BCL11B* (**J**), *SLC17A6* (*VGLUT2*; **K**), *EMX1* (**L**), *TP73* (**M**), *RELN* (**N**), and *GAD2* (**O**). BCL11B, BCL11 Transcription Factor B; EMX1, Empty Spiracles Homeobox 1; EOMES, Eomesodermin; FOXP2, Forkhead Box P2; GAP43, Growth Associated Protein 43; HES1, Hes Family BHLH Transcription Factor 1; SLC17A6, Solute Carrier Family 17 Member 6. SP/IZ, subplate intermediate zone; TBR1, T-Box Brain Transcription Factor 1; TP73, Tumor Protein P73; VGLUT2, vesicular glutamate transporter 2; VIM, Vimentin.

**FIG. 4.**
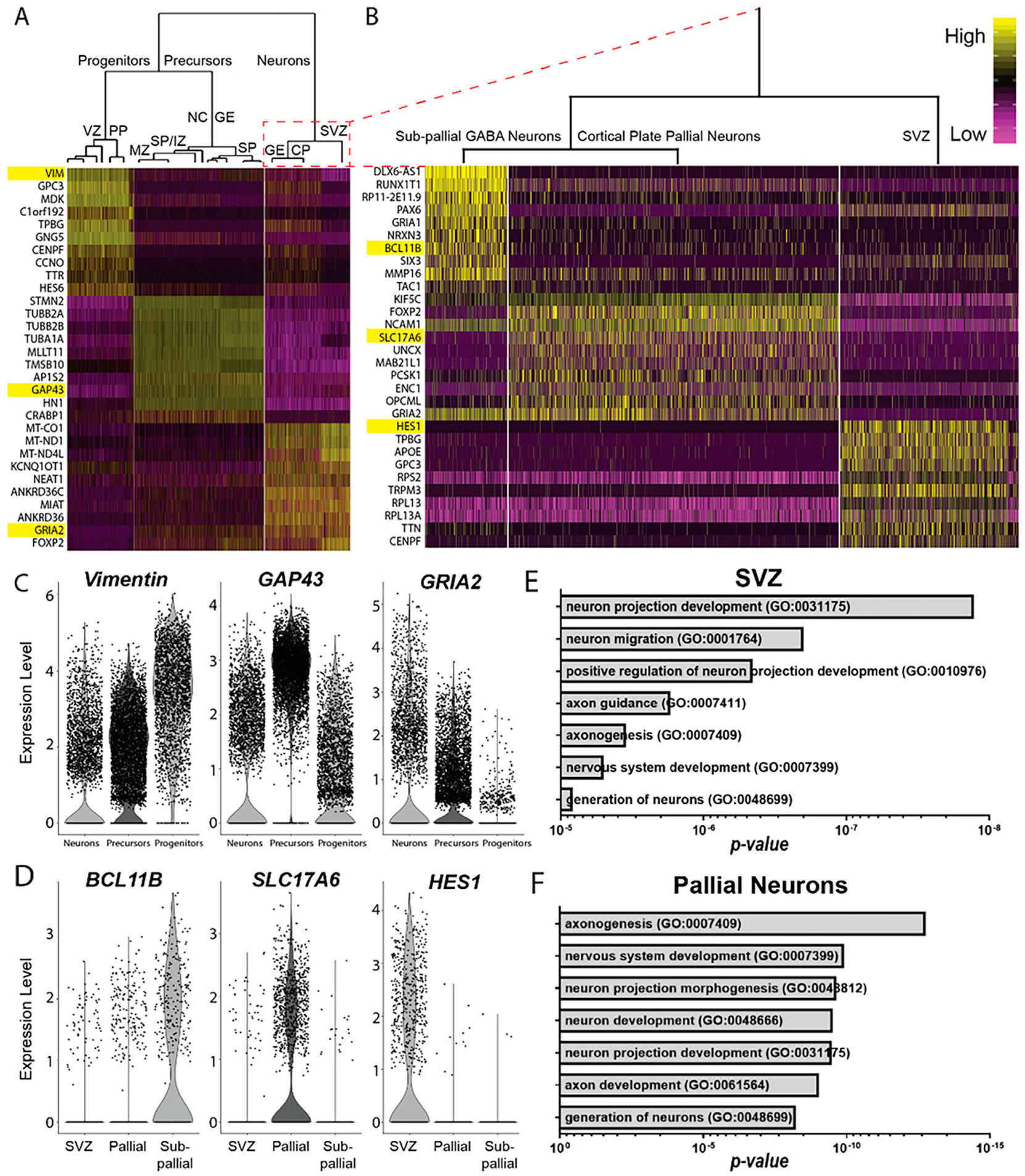
*FEZF2*-expressing cells mature into excitatory glutamatergic neuronal populations. (**A**) PAGODA hierarchical clustering of unbiased populations generated from Seurat tSNE analyses used to delineate the three primary branched clusters with the 10 most differentially expressed genes (DEG) compared in the heatmap. Clusters were identified as “progenitors” (*left*) associated with the VZ and PP; neural “precursors” (*middle*) associated with the MZ, IZ, and SP; and “neurons” (*right*). (**B**) Hierarchical clusters from “neurons” were used to delineate three subclusters comparing the top 10 DEG in the heatmap, which were identified as subpallial GABA neurons (*left*) from the ganglionic eminence (GE), CP pallial neurons (*middle*), and pallial SVZ progenitors (*right*). (**C–D**) Violin plots illustrating specific DEG used to identify the three populations in A (**C**) and B (**D**). (**E–F**) Gene ontology (GO) annotation of terms associated with most significant DEG in either the pallial SVZ (**E**) or excitatory glutamatergic neuronal subgroups from the pallium (**F**).

Data were visualized using tSNE, and unbiased clusters were annotated based on the observed cell type-specific marker expression, including the *FEZF2*-GFP transgene, revealing that most cells expressed appropriate transcriptomic signatures associated with forebrain domains (Figs. 3 and 4, Supplementary Fig. S5). RNA velocity^30^ was used to generate vector-based developmental trajectories by comparing mature spliced transcripts and nascent unspliced isoforms (Fig. 3A-C). Developmental trajectories originating from NEC associated with the ventricular zone (VZ) proceeded in three directions: (1) the sub-ventricular zone (SVZ), (2) the CP, and (3) the PP (Fig. 3A, B). Analogous to early human cortical development, PP vector trajectories split into two directions: (1) the MZ and (2) the SP (Fig. 3A, B). Vector trajectories from the VZ, SVZ, and PP gave rise to distinct IPC associated with the intermediate zone (IZ), which then proceeded to become glutamatergic neurons in the MZ, SP, and CP, with SP cells also directing to the CP (Fig. 3A, B). Analysis of steady-state (spliced) *FEZF2* mRNA levels (Fig. 3C1) compared with nascent (unspliced) transcripts (Fig. 3C2) indicated the rapid splicing of nascent *FEZF2* mRNA and the slow degradation of spliced transcripts (Fig. 3C4). Both unspliced and spliced *FEZF2* transcript expression levels were maintained in RGC (*vimentin* (VIM), *hes family BHLH transcription factor 1* (HES1); Fig. 3D-E), IPC (*eomesodermin* (EOMES); Fig. 3F), and glutamatergic neurons (*T-box brain transcription factor 1* (TBR1), *solute carrier family 17 member 6* (SLC17A6); Fig. 3G, K) associated with the CP (*forkhead box P2* (FOXP2), *BCL11 transcription factor B* (BCL11B); Fig. 3H, J), SP (*growth associated protein 43* GAP43); Fig. 3I), and MZ (*empty spiracles homeobox 1* (EMX1), *tumor protein P73* (TP73), *reelin* (RELN); Fig. 3L-N). SCPN gene expression was present in both the CP and SP domains (Fig. 3G-K). A minor fraction of the cells (~10%) were patterned to the subpallial domains associated with inhibitory GABAergic gene signatures (*glutamate decarboxylase 2* (GAD2); Fig. 3O) and segregated from the pallial domains (Fig. 3B). Taken together, after 3 weeks of *in vitro* differentiation, hiPSC fate specification closely recapitulated gene expression signatures associated with neural cell types at the early stages of forebrain development.

#### FEZF2 cells fate-specify into excitatory glutamatergic neuronal populations.

To further characterize how our directed differentiation paradigm emulates forebrain fate specification, single-cell transcriptomes were analyzed using pathway and gene set overdispersion analysis (PAGODA).^31^ Hierarchal clustering of DEG identified three distinct groups: (1) VZ and PP progenitors; (2) MZ, IZ, SP, neural crest, and subpallial ganglionic eminence (GE) precursors; and (3) SVZ, CP, and subpallial GE neurons (Fig. 4, Supplementary Fig. S5). Group 3 was further subdivided into three distinct subgroups, corresponding to subpallial neurons, pallial CP neurons, and SVZ/IZ progenitors (Fig. 4B-D). GO analysis of the top 100 DEG revealed that SVZ/IZ progenitors exhibited significant enrichment for genes involved in neuronal projection development and neuron migration (Fig. 4E), whereas pallial CP neurons were the most enriched for genes involved in axonogenesis and nervous system development (Fig. 4F). These analyses confirm that hiPSC-derived, *FEZF2*-expressing cells primarily differentiate into excitatory glutamatergic neuronal populations, recapitulating the molecular hallmarks of neuronal fate specification and neurite outgrowth that occur with pallial development.

#### Isolation of fate-committed glutamatergic neurons from FEZF2-GFP cells.

**FIG. 5.**
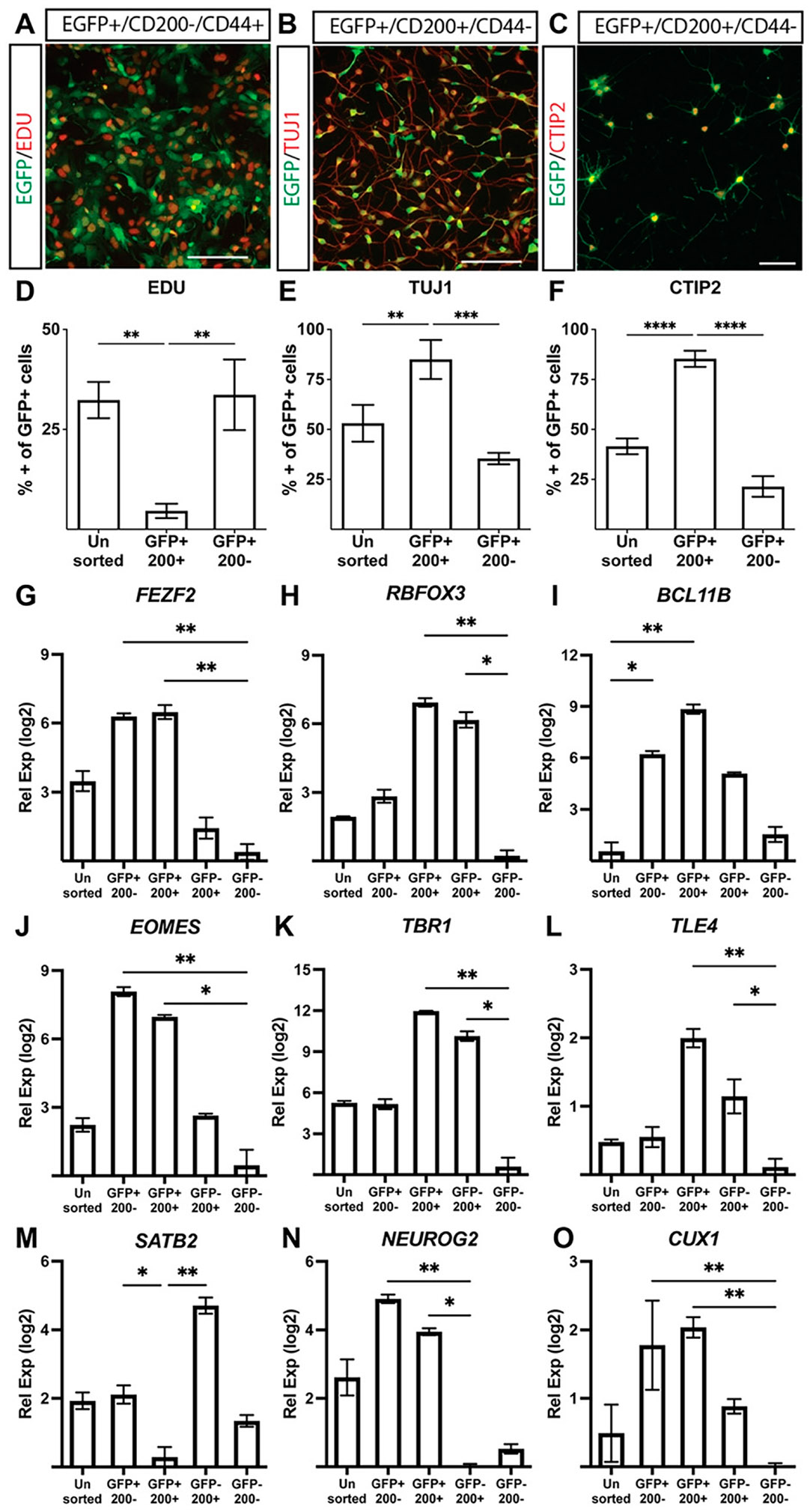
Isolation and enrichment of post-mitotic, FEZF2-EGFP-expressing SCPN. (**A–F**) Multiwavelength fluorescent images and quantification of fixed cells sorted for GFP and CD200 1 day post sort (day 22) and showing co-localization of GFP (*green*) with EdU (**A, D**), TUJ1 (**B, E**), or CTIP2 (**C, F**) compared with unsorted populations. Scale bar = 200 μm. Quantification showing mean ± standard error of the mean (SEM) of unsorted or sorted populations from *n* = 4 independent differentiations using one-way nonparametric ANOVA with Kruskal–Wallis test and uncorrected Dunn’s test. (**G–O**) Transcript quantification of relative gene expression (Rel Exp; ΔΔCt) levels between sorted populations measured for *FEZF2* (**G**), *RBFOX3* (**H**), *BCL11B* (**I**), *EOMES* (**J**), *TBR1* (**K**), *TLE4* (**L**), *SATB2* (**M**), *NEUROG2* (**N**), and *CUX1* (**O**), which were normalized to HPRT (ΔCT) and then within samples to the lowest expressing sample (Log2) mean ± SEM of unsorted or sorted populations from three independent differentiations. Quantification showing mean ± SEM from *n* = 4 independent differentiations using one-way nonparametric ANOVA with Kruskal–Wallis test and uncorrected Dunn’s test. *P < 0.05, **P < 0.01, ***P < 0.001, ****P < 0.0001. 200: CD200. SCPN, sub-cerebral projection neurons.

Using a fluorescence-based antibody screen for surface markers enriched in FEZF2-GFP-expressing cells (Lyoplate, BD Biosciences), we identified CD200 (OX2) as a surface marker enriched in neurons and astroglia precursors.^32,33^ Although they were rarely found in our cultures after 20 days, astroglial precursors were excluded using CD44 (Fig. 5, Supplementary Fig. S7).^32-36^ The sequential generation of neuronal subtypes from a multipotent progenitor pool is created when a neural progenitor exits the cell cycle and commits to a fate-specified neuronal subtype.^37^ Analyzing their proliferative potential, sorted CD200+ cells displayed significantly lower EdU incorporation compared with unsorted cells or GFP+/CD200− cells (Fig. 5A, D). GFP+/CD200+ neurons also showed the robust co-expression of TUJ1, a neuron-specific tubulin, compared with GFP+/CD200− cells (Fig. 5B, E). Enrichment for *BCL11B* (CTIP2), a marker of post-mitotic SCPN, was observed in GFP+/ CD200+ cells compared with GFP+/CD200− populations (Fig. 5C, F). Although RT-qPCR analysis revealed no difference in *FEZF2* expression between GFP+/CD200+ and GFP+/CD200− populations (Fig. 5G), sorted GFP+/CD200+ cells exhibited significant enrichment for SCPN-associated genes, including *BCL11B, TBR1*, and *TLE4* (Fig. 5I, K, L). *RNA binding fox-1 homolog 3* (RBFOX3) (NEUN) was similarly enriched in GFP+/CD200+ cells and GFP−/CD200+ cells (Fig. 5H). These results indicate that sorting for FEZF2-GFP and CD200 provides a robust method to enrich for post-mitotic, fate-committed SCPN.

### Sorted FEZF2-GFP+ cells exhibit spontaneous electrophysiological activity

**FIG. 6.**
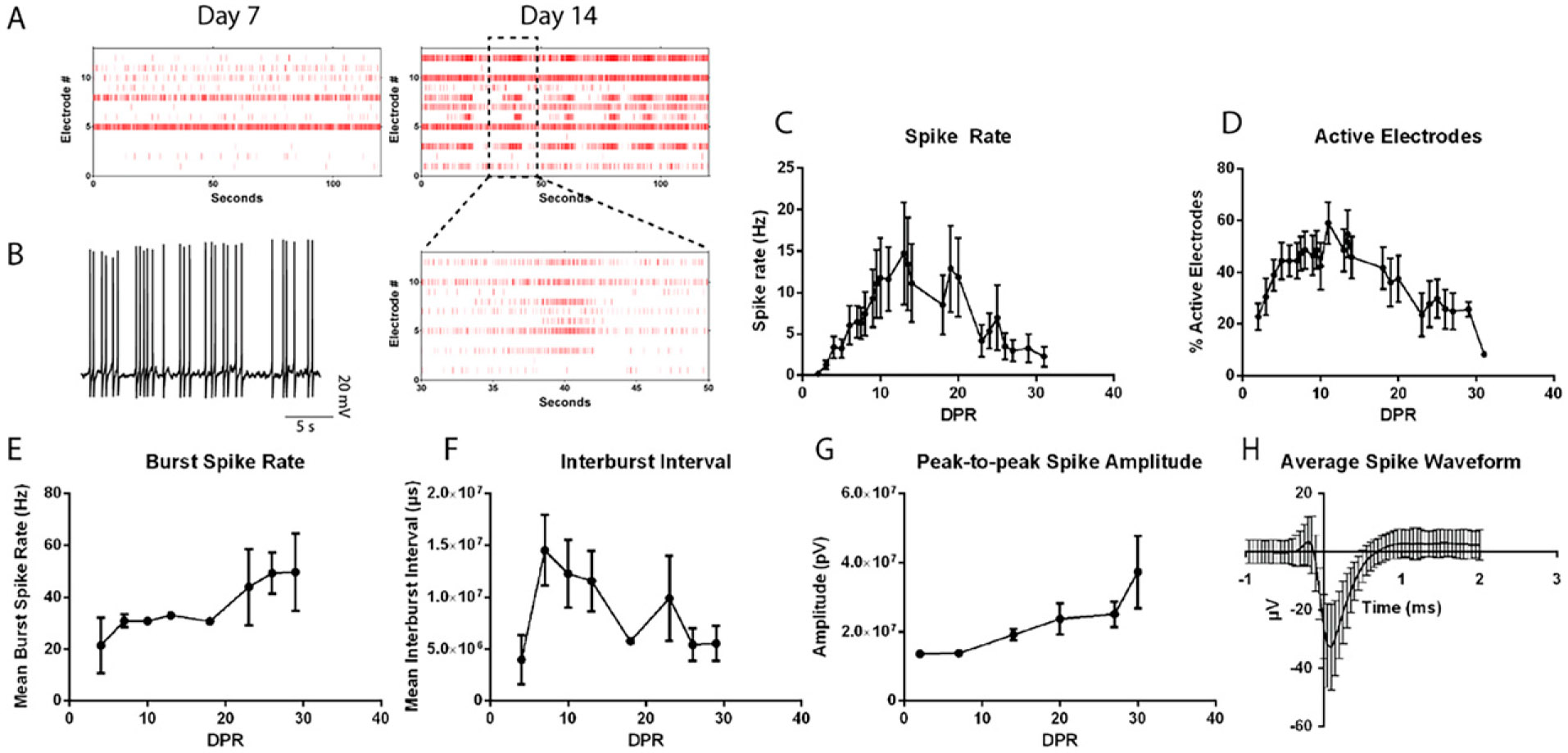
FEZF2-EGFP+ cells exhibit mature electrophysiological activity associated with SCPN. (**A**) Raster plots of spikes detected on 12 individual electrodes (*y*-axis) over 2 min (*top two*) and 20 s (*bottom*). Recordings from the same well are shown at 7- and 14-days post-replating (DPR). (**B**) Example trace of a spontaneous action potential measured by whole-cell patch clamp. (**C–G**) Measurements from 2-min recordings of isolated SCPN in one MEA plate of 12 wells over 31 DPR. (**C**) Average spike rate from active electrodes, mean ± SEM. (**D**) Average percent of active electrodes per well, mean ± SEM. (**E**) Average burst spike rate of three recordings, mean ± SEM. (**F**) Average inter-burst interval (IBI) of three recordings, mean ± SEM. (**G**) Average spike amplitudes (pV), mean ± SEM. (**H**) Example of spike cutouts from an average of 500 spikes in a single well from a single electrode as μV over a 3-ms duration from isolated SCPNs, mean ± SEM.

To track the progression from single spikes to spike trains and ultimately bursting patterns with neuronal network maturation,^38^ MEA recordings measuring extracellular activity were performed on sorted GFP+/CD200+ SCPN over 4 weeks. Spontaneous spike activity was observed at 1 DPR, followed by spike trains after 7 DPR and synchronous burst firing after 14 DPR (Fig. 6A). Parallel patch-clamp recordings at 14 DPR confirmed these spikes as spontaneous neuronal action potentials (Fig. 6B). The spike rate (Fig. 6C) and percentage of active electrodes (Fig. 6D) peaked between 10 and 21 DPR. The burst spike rate (Fig. 6E) and spike amplitude (Fig. 6G) continued to increase, whereas the inter-burst interval (Fig. 6F) continued to decrease over 4 weeks. FEZF2-GFP+ neurons exhibited stereotypical spike waveforms associated with SCPN functional maturation (Fig. 6H). These findings indicate that FEZF2-GFP+ putative SCPN exhibit action potentials immediately after sorting and continue to mature into synchronized burst-firing neuronal networks within 4 weeks.

### Spinal motor neurons enhance the viability and functional properties of SCPN

**FIG. 7.**
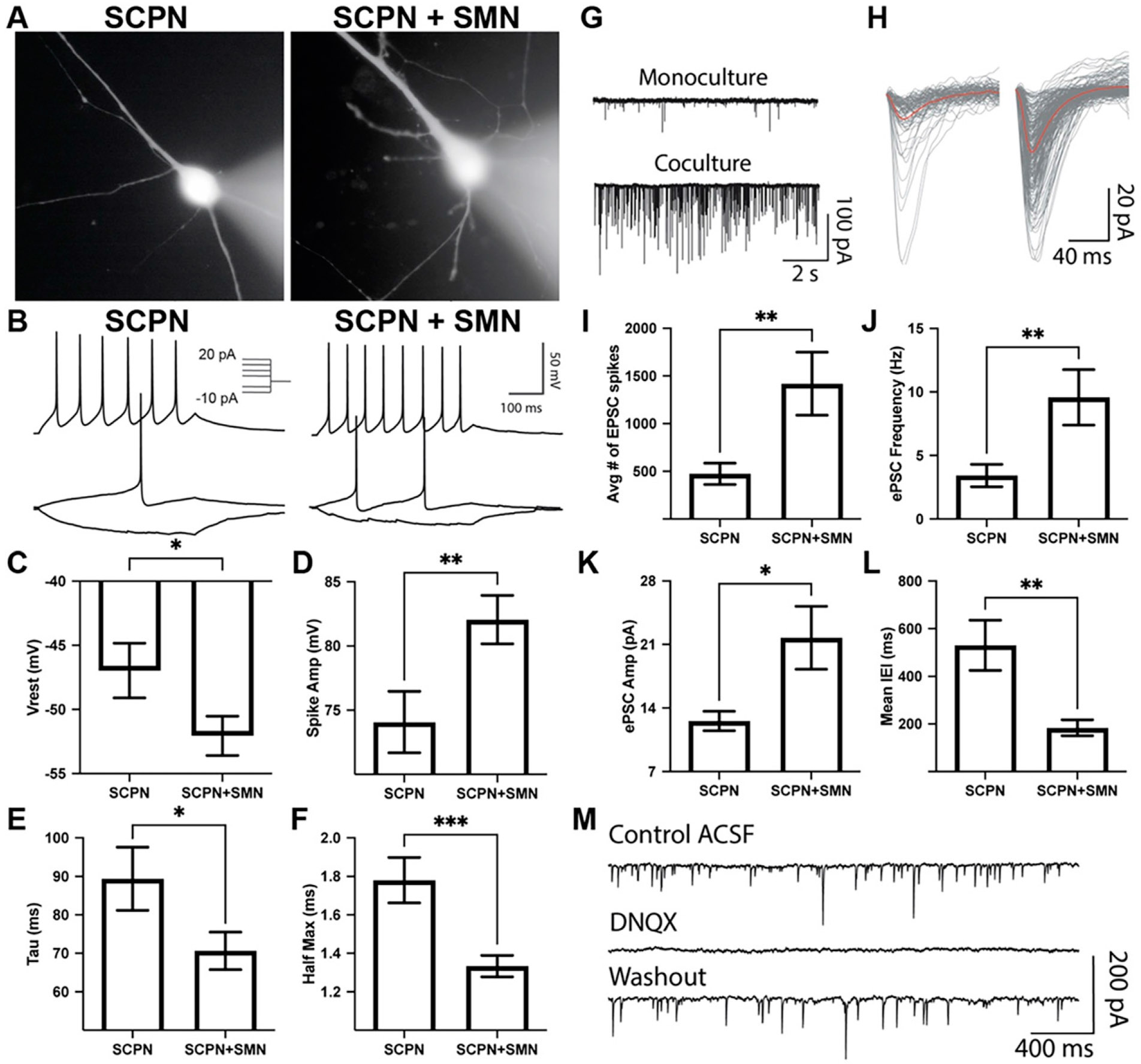
Putative FEZF2-EGFP+ SCPN exhibit electrophysiological properties and synaptic activity that are enhanced by spinal motor neurons (SMN) co-culture. Patch-clamp recordings were performed 4 weeks after FACS isolation. (**A**) FEZF2-EGFP+ cells filled with biocytin-555 exhibit pyramidal neuron morphology. (**B**) Depolarizing current injections reveal an absence of spike frequency adaptation in both conditions. (**C–F**) SCPN + SMN co-culture enhanced the maturation of functional properties, as shown by decreased resting membrane potential (**C**), increased spike amplitude (**D**), decreased Tau time constant (**E**), and decreased half-max (**F**). Statistical significance was quantified from *n* = 28 cells (SCPN) and *n* = 40 cells (SCPN + SMN) using the Mann–Whitney U test. (**G**) Raw traces of spontaneous excitatory post-synaptic current (EPSC) activity from SCPN (*top*) or SCPN + SMN (*bottom*) at a holding potential of −60 mV. (**H**) Several individual EPSC spikes (*gray*) overlaid with the mean trace (*red*). (**I–L**) SCPN + SMN co-culture enhanced synaptic activity, as shown by the increased average number of EPSC (**I**), increased EPSC frequency (**J**), increased EPSC amplitude (**K**), and decreased inter-EPSC interval (IEI) (**L**). Statistical significance was quantified from *n* = 11 cells (SCPN) and *n* = 18 cells (SCPN + SMN) using the Mann–Whitney U test. (**M**) Raw trace from SCPN monoculture of spontaneous ePSC before (*top*), during bath application of 20 μM of DNQX (*middle*), and after washout (*bottom*). N = 5 differentiations. *P < 0.05, **P < 0.01, ***P < 0.001. DNQX, 6,7-dinitroquinoxaline-2,3-dione disodium salt.

Corticospinal motor neurons (CSMN), which are a specialized subset of SCPN, navigate their axons into the spinal cord to synapse onto SMN.^39^ To investigate this interaction, we co-cultured hiPSC-derived SCPN with hiPSC-derived SMN. After 3 weeks of differentiation using a modified SMN protocol,^40^ GFP−/CD200+/CD44− SMN were FACS-isolated and enriched for cholinergic neuronal markers (Supplementary Fig. S6). FEZF2-GFP+ SCPN were replated either alone or in co-culture with FACS-isolated SMN to investigate their effects on SCPN electrophysiological properties via whole-cell patch-clamp recordings (Fig. 7A). Comparison of evoked action potentials between putative SCPN in monoculture and co-culture revealed no differences in input resistance and the spike threshold (Supplementary Table S2), with both conditions exhibiting nonadaptive action potentials (Fig. 7B). SCPN in co-culture with SMN exhibited significantly decreased resting membrane potential, increased spike amplitude, decreased membrane time constant Tau, and decreased half max when compared with SCPN alone (Fig. 7C-F). Therefore, SCPN co-culture with SMN enhanced several electrophysiological parameters associated with functional maturation.

When measuring spontaneous excitatory post-synaptic currents (EPSC), SCPN co-culture with SMN exhibited significantly increased EPSC frequency, increased EPSC amplitude, and reduced inter-event interval (Fig. 7G-L). To confirm the contribution of α-amino-3-hydroxy-5-methyl-4-isoxazolepropionic acid (AMPA) receptors to EPSC recordings, 6,7-dinitroquinoxaline-2,3-dione disodium salt was applied, leading to the loss of spontaneous EPSC activity that recovered after washout (Fig. 7M). Taken together, SCPN co-culture with SMN enhances the strengthening of synaptic inputs and faster synaptic integration, resulting in more precise and efficient excitatory neurotransmission.

**FIG. 8.**
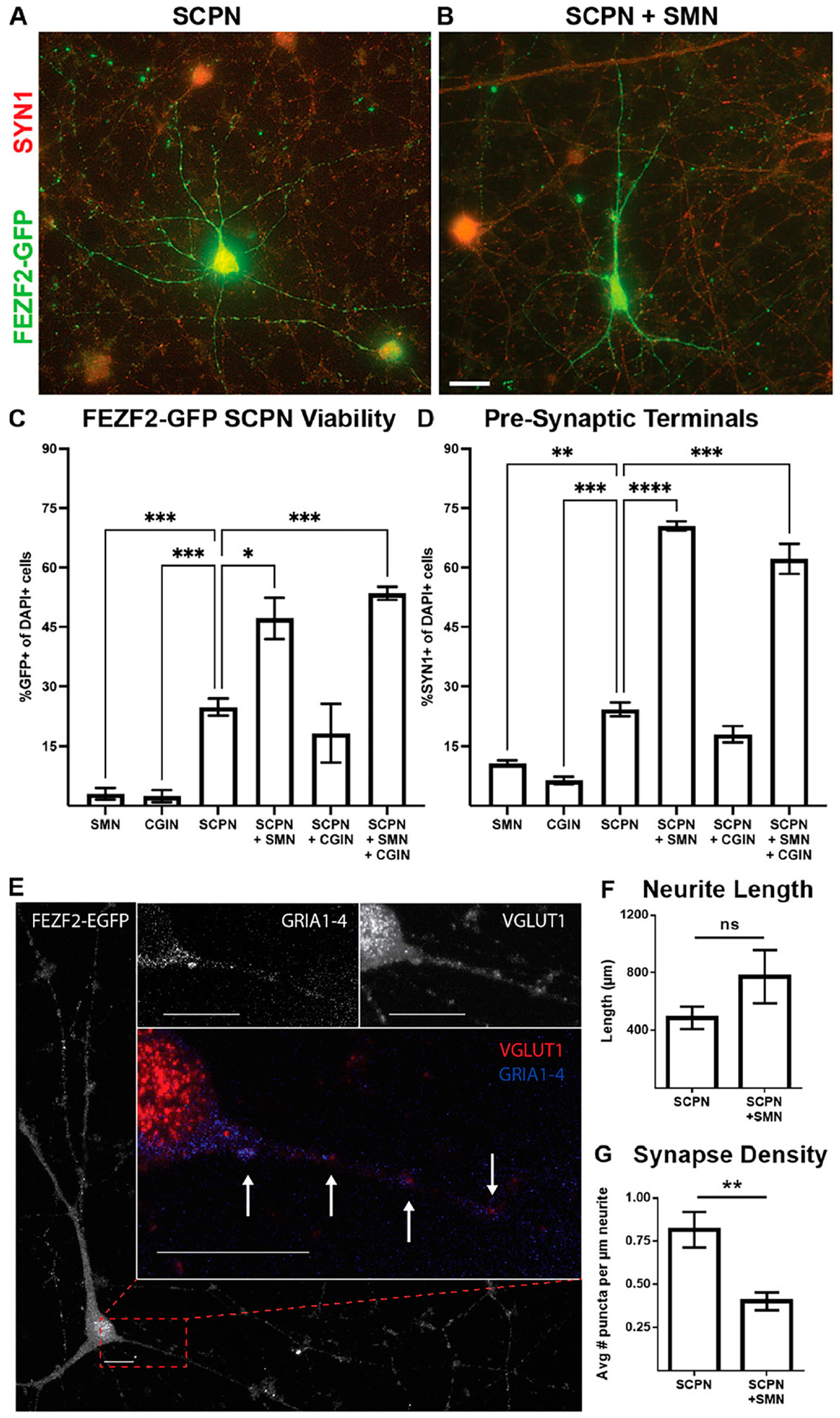
SMN co-culture increased SCPN viability and formation of presynaptic terminals while reducing the number of glutamatergic synapses. (**A–D**) Images and analyses from six conditions were performed after 4 weeks of *in vitro* cell culture: (1) SMN monoculture, (2) cortical GABAergic interneurons (CGIN) monoculture, (3) FEZF2-GFP+ cells (SCPN) monoculture, (4) SCPN + CGIN, (5) SCPN + SMN, or (6) SCPN + SMN + CGIN co-cultures from *N* = 3–5 differentiations. FEZF2-GFP (*green*) and the presynaptic terminal protein synapsin1/2 (SYN1; *red*) were both expressed in SCPN (**A**) and SCPN + SMN (**B**) conditions. (**C–D**) Quantification of FEZF2-GFP+ (**C**) and SYN1+ (**D**) shown as a percentage of DAPI+ cells were significantly enriched in all co-culture conditions containing SMN. Statistical significance was determined using unpaired *t*-test with Welch’s correction vs. SCPN monoculture conditions. *P < 0.05, **P < 0.01, ***P < 0.001, ****P < 0.0001. (**E**) Confocal image of SCPN + SMN co-culture showing colocalization of presynaptic terminal protein VGLUT1 (*red*) and post-synaptic terminal protein GRIA1-4 (*blue*) ICC staining on FEZF2-EGFP (*green*) neurite 63× oil immersion objective and 10-μm scalebar. (**F**) Quantification of the density of glutamatergic synapses (i.e., VGLUT1+ and GRIA1-4+) per μm of GFP+ neurite exhibited a reduction in the SCPN + SMN condition when compared with SCPN monoculture. Unpaired Student’s *t*-test with *n* = 10 cells measured for both SCPN and SCPN + SMN conditions (*P* < 0.005).

Previous studies have shown that an excess number of SMN produced in the embryo will be pruned down to only those that successfully synapse onto their muscle targets.^41,42^ Neurotrophic factors from target cells promote the survival of motor neurons that successfully innervate their target cells, while target-deprived motor neurons undergo programmed cell death.^43,44^ To assess the survival enhancement conferred by providing the CSMN target population, we co-cultured our putative SCPN with hiPSC-derived SMN and/or hiPSC-derived CGIN in parallel cultures.^16^ Immunofluorescence microscopy analysis after 4 weeks of culture revealed that putative SCPN exhibited increased survival when co-cultured with SMN, regardless of the presence of CGIN (Fig. 8A-C). Furthermore, synapsin 1, a marker of presynaptic terminals, was enriched in SCPN conditions in co-culture with SMN (Fig. 8D).

To further explore synapse formation, confocal imaging was used to visualize neuronal somas and processes (Fig. 8E). Synaptic puncta, co-localization of presynaptic vesicular glutamate transporter 1 and postsynaptic pan-AMPA receptors (GRIA1–4), revealed half the glutamatergic synapse density in SCPN + SMN co-cultures (Fig. 8F-G). Together, these findings indicate that hiPSC-derived FEZF2-GFP+ SCPN demonstrate increased survival and enhanced glutamatergic electrophysiological activity while in co-culture with SMN, although not through an increased quantity of glutamatergic synapses.

## Discussion

Several directed differentiation protocols have been used to pattern hiPSC into forebrain domains that appear to retain an intrinsic maturation rate with pallial cell fate specification.^6,16,45-47^ The current *in vitro* differentiation strategies show the peak generation of neurons with markers of deeplayer cortical subtypes such as TBR1 and CTIP2 from 30 to 60 days or longer.^20-22^ We sought to enhance neuronal specification from hiPSC-derived forebrain progenitors with three small-molecule inhibitors of signaling pathways that regulate cell division and self-renewal (Supplementary Fig. S2). Previous studies have used similar combinations to enhance neuronal specification toward nociceptors,^48^ glutamatergic neurons,^24^ midbrain dopamine neurons,^49^ and SMN.^50^

When comparing different mammals, the generation of increased numbers of IPC correlates with increased cortical complexity and size.^51^ Lineage tracing and real-time imaging approaches have shown that cortical NEC divide exclusively in a symmetric mode before neurogenesis and then become RGC, which divide increasingly in an asymmetrical mode, giving rise to other RGC and either a neuron or an IPC.^52,53^ It remains unclear from our experiments what fraction of the glutamatergic neurons results directly from RGC prematurely exiting the cell cycle and what fraction results indirectly from increased IPC.^54,55^ Upon the loss of FGF signaling, there is an initial increase in the numbers of newly born IPC and neurons in mice,^56^ although FGFR1 inhibition alone was not sufficient to generate neurons in our studies (data not shown). Therefore, our combined small-molecule treatment enhances the production of IPC and glutamatergic neurons at the expense of the NEC and RGC stages of cortical development.

Most hiPSC forebrain directed differentiation protocols generate a mixed population of cells at varying stages of development with limited electrophysiological function.^15^ Differentiated organoid systems could be employed to distinguish the morphology of multipolar IPC from bipolar radial glia associated with the SVZ and the unipolar morphology of outer RGC,^54^ but these systems display low fidelity in generating NEC and early RGC types.^6^ Studies in mice showed that multipotent FEZF2+ RGC generated all major glutamatergic projection neuron subtypes as well as astrocytes and oligodendrocytes in accordance with the classical model of cortical neurogenesis.^18,57^ Two-dimensional cortical cultures lack apical (ventricular) and basal (pial) orientations, and thus, the identification of cell types must be made by molecular identity. We used RNA velocity analysis to infer temporal dynamics in our single-cell gene expression data.^30,58^ These relationships are illustrated as a continuum of vectors between cell types identified by genes observed in analogous scRNA-seq data from the human fetal neocortex.^3,6,59^ The breadth of developmental states in an unsorted differentiation contextualizes the mapping of this continuum, which revealed *FEZF2*-expressing subpopulations throughout neuronal subtype specification (Fig. 3A, B). Our analyses showed that *FEZF2*-GFP+ NEC gave rise to multiple clusters of *FEZF2*-GFP+ RGC. We did not observe *HOPX1*+ outer RGC,^55^ but RGC then specified into separate clusters of IPC (Fig. 3A, B). Of particular interest, *FEZF2*-GFP+ IPC gave rise to glutamatergic neuronal subgroups in clusters associated with distinct developmental regions: CR neurons, SP neurons, and CP neurons (Fig. 3). CR neurons, located in the MZ and hem, secrete the extracellular protein RELN, involved in the regulation of radial neuron migration.^60-62^ The SP is defined as a transient zone below the CP and plays a key role in neural circuit formation and cortical self-organizing processes in early developmental stages.^63,64^ Early studies in the human fetal cortex suggested the possibility that CR neurons of the MZ may arise locally in the VZ of the dorsal cortex or by PP splitting.^27,65^ SP neurons are generated from TBR2 (EOMES)-expressing IPC located in the SVZ.^66^

Our FEZF2-GFP reporter hiPSC line gives a real-time graded evaluation of *FEZF2* expression, which allowed us to optimize culture conditions that enhanced SCPN specification. Cell sorting combining FEZF2-GFP+/CD200+/CD44− allowed for the enrichment of post-mitotic, putative SCPN that enriched for *FEZF2*, CTIP2, *NEUN*, and TUJ1 (Fig. 5B-D). Although our sorting strategy enriched for the molecular markers of CSMN, we also observed enrichment for the genes expressed in SP neurons, including *TBR1, TLE4, NEUROG2*, and *ETS variant transcription factor 1* (Fig. 5K-L, Supplementary Fig. S1B).

We wanted to test whether our putative CSMN would integrate into a motor circuit. In rodents, CSMN synapse onto interneurons to initiate SMN activity.^67,68^ However, in primates and especially humans, the majority of CSMN connect monosynaptically onto cholinergic SMN, with collaterals and subpopulations additionally targeting spinal GABAergic interneurons.^69,70^ Given that cholinergic neurons in the brainstem and striatum are targets for SCPN,^71,72^ we chose CGIN as a negative control in our co-culture experiments. CGIN reside alongside CSMN in the cortex, and, while not cholinergic, differentiations include a small population of basal forebrain cholinergic neruons^16^; moreover, both cell types are nontarget populations for CSMN. When co-cultured with SMN, putative CSMN exhibited enhanced electrophysiological maturation, glutamatergic synaptic activity, and survival, which were not observed in co-culture with CGIN (Figs. 7 and 8). Electrophysiological studies from the mouse cortex show that corticocortical neurons exhibit adapting spike activity, while corticofugal neurons show nonadapting spike activity.^17,73^ Therefore, nonadapting spikes observed in FEZF2-GFP+ cells suggest electrophysiological characteristics indicative of putative CSMN (Fig. 7B).

Taken together, these results identify FEZF2-GFP expression in multipotent pallial progenitors, whose laminar fate restriction extends to glutamatergic projection neuronal subgroups in the CP, SP, and MZ. Our FEZF2-GFP hiPSC line will expand the knowledge of progenitor cell heterogeneity within the neocortex and help uncover mechanisms that generate projection neuron diversity. Future studies will be aimed at the detailed characterization of RGC, IPC, CR neurons, and SCPN in multiple hiPSC lines and in threedimensional organoids to provide more stringent *in vitro* readouts of *in vivo* human cortex development.^6^

## Supplementary Material

supplement

Supplementary Data
