# Supplementary material for: The Specification and Functional Maturation of Sub-Cerebral Projection Neurons Derived from Human Induced Pluripotent Stem Cells
Source: Stem Cells Dev. Author manuscript; Available in PMC 2026 May 12. (PMC13162234; doi:10.1177/15473287251399623)
Supplement: supplement [file NIHMS2168150-supplement-supplement.pdf]

Supplemental Methods

Maintenance and expansion of Human Pluripotent Stem Cells

Human iPSCs were maintained under feeder-free conditions on tissue culture treated 6-well plates (Olympus Cat. 25-105MP) with Geltrex LDEV-Free hESC-Qualified Reduced Growth Factor Basement Membrane Matrix (Life Technologies) in StemFlex™ Medium (Life Technologies) containing Primocin (InvivoGen) at 37°C with 5% CO_2 and 21% O_2. Stem cells were passaged for expansion and maintenance at <90% confluence by manual picking or chemical dissociation using EDTA (100 µM; GIBCO) and were used at passage 40 or lower. The 18a cell line, derived from a healthy 48-year-old female, and the 11a cell line, derived from a healthy 37-year-old male, were obtained from the Harvard Stem Cell Institute and were approved for use on this project by the University of Texas at San Antonio. These cell lines were reported to have normal karyotypes[1,2], determined by Nanostring nCounter Human Karyotype Panel (Nanostring Technologies, USA). In addition, each cell line tested negative for mycoplasma with a MycoAlert Mycoplasma Detection Kit (Lonza, LT07-218).

Generation and validation of stable FEZF2-GFP hiPSC lines

A transcription activator-like effector nuclease (TALEN) was used as described previously[3] to accurately target the FEZF2 genomic locus in hiPSC line 18a, a non-diseased control[2]. We targeted the 3' end of the FEZF2 gene because we wanted to maintain endogenous FEZF2 gene expression while also preserving potential enhancer domains at the 5' end. The N-terminal of the FEZF2 protein contains an engrailed homology domain (EH1 domain), which is known to recruit the transducin-like enhancer

of split (TLE) family transcriptional co-repressors (Hashimoto et al., 2000). The left and right TALEN pairs together with the HR construct were transfected into a single cell suspension of 2x10^6 cells (Neon Transfection system; ThermoFisher). After transfection, the cell suspension was plated into one well of a six well plate containing 3 mL of pre-warmed mTesR media with 10 µM Y27632 and incubated at 37 °C with 5% CO_2. After 72 hours, puromycin selection was added to the mTesR media for 10 days. Puromycin resistant colonies were picked and validated by PCR for integration at the 5' and 3' ends using primers outside of the 700 bp homology arms in conjunction with primers to the transgene insertions. Primers used were P1-TAGCCAAGCGTCGCCTTTTT; P2-CTTGTGGCCGTTTACGTCG; P3-CAAGAAGCCTTTCACGTGCG; 1439.rev (P4)-CTGGGAATGATTAGATGATTAATSTAATSCTSTTTGAAGGGTCTCTOTTTTCTCTOTTAATTTGGGTCATTACTTAGAAAGCAAGCGTGAGATCATTTCA. Transiently transfected CRE recombinase was used to excise the PGK puromycin resistance cassette. Cells which survived hygromycin selection were validated by the absence of the PCR product using a primer targeted within the puromycin resistance gene in conjunction with the previous 3' primer targeted to the region of the chromosome downstream of the endogenous FEZF2 gene and outside of the 700 bp homology arms. Additionally, clonally expanded parallel cultures treated with puromycin proved inviable, further validating excision of the puromycin resistance cassette. Further validation was performed with a set of PCR primers targeted ~120 bp 5' upstream and 3' downstream of the endogenous FEZF2 stop codon to detect an insertion of the length of the P2A EGFP sequence.

Fluorescent activated cell sorting

On differentiation days 20-30, cell culture medium was collected and replaced with Accutase (Stem Cell Technologies) for a 30–60-minute dissociation. Cell suspensions were collected with gentle trituration and diluted in tubes containing the collected culture medium. Cell suspensions in media were then centrifuged at 211 x g at ambient temperature for 5 minutes to pellet the cells. Cell pellets were resuspended in fresh Neurobasal Medium (NBM) with Y27632 (10 µM; Tocris), filtered through a 70 µm cell strainer, and cells counted in a Countess II automated cell counter. Viable cells were identified by Trypan blue exclusion. Staining was performed with 100 µl of staining solution consisting of NBM with Y27632 and a combination of anti-CD200-APC (10 µl per million cells) and anti-CD44-PE (2 µl per million cells) (Table S1). Staining was performed for 30 minutes on ice in the dark. Dead cell positive controls were produced by heating cells to 55°C for 15 minutes. Next, stained cell suspensions were washed with excess medium, pelleted at 211 x g at 4°C for 5 minutes, and resuspended in sorting buffer consisting of PBS and 0.9% HEPES, 1% BSA, 0.2% EDTA, and 0.2% Primocin.

Cells were sorted on a FACSAria II (BD Biosciences) by first gating for intact cells by forward scatter area and side scatter area, then gating for single cells by forward scatter width and forward scatter height, and again for single cells by side scatter width and side scatter height. Cells were then sorted for EGFP positive and negative by FITC and side scatter area, using the 11a non-transgenic hiPSC line differentiated in parallel as a GFP-negative control. Subsequently, neurons were sorted from glial progenitors by CD200+/CD44-. Groups of EGFP+/CD200+/CD44- or EGFP-/CD200+/CD44- were collected in NBM or Brain Phys Medium with Y27632 (Tocris) and Forskolin (10 µM),

Ascorbic Acid (200 nM), BDNF (10 ng/ml), GDNF (10 ng/ml), and Neurotrophin 3 (10 ng/ml) (NBM +FABG3 +Y27632). Post sort analysis was processed using FlowJo v10.7.1 (Tree Star). Following FACS, collected cells were centrifuged at 211 x g in 4°C for 5 minutes to pellet the cells, counted with trypan blue in a Countess II, and plated for downstream experiments on Geltrex coated wells in NBM +FABG3 +Y27632 overnight and maintained in NBM +FABG3.

Single-cell RNA-sequencing

10x Genomics single-cell libraries were generated by the University of Texas at San Antonio (UTSA) and Albert Einstein College of Medicine Genomics Cores. Briefly, cell suspensions were loaded into Chromium microfluidic chips with 3′ gene-expression v3 chemistry and used to generate single-cell gelbead emulsions (GEMs) with the Chromium controller (10x Genomics) per manufacturer recommendations[4]. Sufficient cells were loaded to target 10,000 single cells captured per sample were targeted for capture in GEMs. Libraries were prepared following manufacture recommendations and were subsequently sequenced on NextSeq 500 and NovaSeq instruments (Illumina) with PE150 chemistry. Trimmed FASTQs (26bp Cell barcode and UMI Read1, 8bp i7 index, and 91bp Read2), were generated using CellRanger mkfastq (10x Genomics) and subject to primary analysis (alignment, filtering, and UMI counting) to determine gene transcript counts per cell quality control, clustering and initial statistical analysis with CellRanger count (10x Genomics) and a custom GRCh38 (human hg38) reference genome containing the EGFP cDNA sequence.

Raw count matrices were subsequently imported to Seurat[5,6], filtered for high-quality data (cells expressing ≥ 800 detected genes, genes expressed in ≥ 3 cells) and gene

expression values were log normalized and scaled. For comparisons with fetal tissue data sets, integrated cell clustering was performed using a K-Nearest- Neighbor-Pooling Matching (KNNPM) method and harmony-corrected principle components were used to construct the integrated UMAP. Clusters were manually assigned cell types based on canonical marker gene expression, including the ones shown in figure 2B. These cell type assignments were further validated for consistency between hiPSC-derived cells and fetal cells using groups of highly cell type-specific genes based on Wilcoxon rank-sum tests to create cell type module scores with Seurat. The module groups shown in figure 2C include MZ (*RELN*, *TP73*, *PGF*, *CDKN1A*, *CCNO*, *AC073114.1*), CP (*NEUROD2*, *NEUROD6*, *PPP1R1B*, *SLC17A7*, *SLC24A2*, *SLA*, *BCL11B*, *SATB2*), PP/SP (*EBF1*, *TBR1*, *EBF2*, *SLC17A6*, *EBF3*, *SAMD5*, *SAMD3*, *NEFM*, *NEFL*, *MAB21L1*, *UNCX*, *LHX5*, *LHX1*), VZ/SVZ (*HES1*, *GLI3*, *PAX6*, *VIM*, *SOX2-OT*, *HES5*), IN/LGE (*MEIS2*, *ISL1*, *EBF1*, *SP8*, *SIX3*, *PBX3*, *SHZ1*, *ZNF503*, *GAD2*, *GAD1*, *SLC32A1*, *DLX6-AS1*, *DLX1*, *DLX2*, *DLX5*, *DLX6*, *ARX*, *NRXN3*), Hem (*WNT3A*, *LMX1A*, *RSPO2*, *RSPO1*, *FYB2*, *OTX2-AS1*, *GJA1*), IZ (*EOMES*, *NEUROD4*, *NEURODG2*, *PPP1R17*, *NEUROD1*, *NHLH1*). To construct the Pearson correlation coefficient heatmaps, each sample group was randomly downsampled to include the same number of cells from each integrated cell type so that the comparisons would not be biased based on cell type proportion or number of cells. Code for generating figures 2, S3-4 is available at https://github.com/Sesukai87/Fezf2-Study. Figures 2 and S3 and S4 were created with Biorender.com.

For Velocity analysis, unsupervised cell clustering and tSNE analyses were performed in Seurat based on the statistically significant principal components. The top-10 and -100 differentially expressed genes (marker genes) of each cell cluster were determined by log

fold change ≥ 0.25 using a default Wilcoxon rank-sum test. Cell clustering was performed using a K-Nearest-Neighbor-Pooling Matching (KNNPM) method and projection/visualization (tSNE plots) RNA velocity was performed with velocyto.R[7,8] with default parameters after filtering cells with fewer than 1800 genes, and genes with fewer than 10 reads and detected in only five or fewer cells. Unbiased hierarchical clustering was performed using the Pagoda 2 web application[9]. Gene ontology (GO) Analysis was performed using Enrichr[10,11].

Immunofluorescent staining

Cells were fixed using PBS with 4% paraformaldehyde for 15-20 minutes, washed three times with PBS, neutralized using PBS with 100 mM glycine for 5 minutes, washed three times with PBS, incubated with primary antibodies overnight at 4°C in a blocking solution of PBS with 5% donkey serum and with 0.3% Triton-X (Sigma) for permeabilization, and then washed three times with PBS. Incubation with secondary antibodies was performed at room temperature for 1-2 hours with blocking solution, washed three times with PBS, counterstained for nuclei with Hoechst 33342 (sigma, 23491-52-3) or DAPI (ThermoFisher, 62248) for 5 minutes, and then washed three times with PBS. Coverslips were mounted with Diamond Antifade. Antibodies used are listed in Table S1. Edu staining was performed with Click-iT EdU Cell Proliferation Kit for Imaging, AF647 dye (Thermo, C10340) according to the manufacturer instructions.

Photomicroscopy and Image Quantification

Cell quantification was performed on the entire surface of individual coverslips. Images were captured by an EVOS 3000 at 10X magnification. Raw image files were imported

into Cell Profiler™ to quantify cell counts and fluorescence intensities. Hoechst or DAPI stained nuclei were counted as individual cells, from which a watershed algorithm was used to identify cell bodies. Cells were binned as EGFP+ if their median pixel intensity exceeded the average +1 standard deviation of all cells from the LMN and CGIN alone conditions (without EGFP-expressing cells). The number of EGFP+ cells were normalized to total Hoechst- or DAPI-positive nuclei and expressed as percent positive. Quantifications of technical replicates were averaged together to count as one biological replicate (all tissues derived from a single differentiation). Three or more biological replicates were used for statistical analyses in Graph Pad Prism™. An unpaired Student's T-test, assuming Gaussian distribution and the same SD was used to compare two groups and an ordinary one-way analysis of variance was used for more than two groups. Statistical significance was established by p-value: * $p<0.05$, ** $p<0.01$, ***$p<0.001$ Representative images were processed using Adobe Photoshop and assembled into figures using Adobe Illustrator.

Quantitative real time PCR

Relative transcript abundance of hiPSCs or 21 day differentiated sorted or unsorted cultures were measured via quantitative reverse transcription PCR (qRT-PCR), with cycle threshold (CT) values of each gene normalized first to an internal control gene and second within samples to the lowest expressing sample (Log2) mean ± standard deviation (SD) for n=3 samples, and compared using an ordinary one-way ANOVA with Dunnett's multiple comparisons test. Internal control genes were *Glyceraldehyde-3-Phosphate Dehydrogenase* (*GAPDH*) for FEZF2 and EGFP and *Hypoxanthine Guanine Phosphoribosyltransferase* (*HPRT*) for *BCL11B*, *ETV1*, *RBFOX3*, *SATB2*, *TBR1*, and

TLE4. PCR TaqMan probes purchased from ThermoFisher were FEZF2: Hs01115572_g1 FAM, CTIP2 (*BCL11B*): Hs01102259_m1 FAM, ER81 (*ETV1*): Hs00951951_m1 FAM, NEUN (*RBFOX3*): Hs01370653_m1 FAM, *SATB2*: Hs00392652_m1 FAM, *TBR1*: Hs00232429_m1 FAM, *TLE4*: Hs00419101_m1 FAM, *GAPDH*: Hs02758991_g1 FAM, eGFP: Mr04329676_mr VIC-MGB, and *HPRT1*: 4326321E VIC-MGB. qRT-PCRs were run on a Bio-Rad CFX96 Touch Real-Time PCR System using 20uL reactions with 3-4 technical replicates and 3-5 experimental N. The program ran 95°C for 2 minutes, 95°C for 10 seconds, then 60°C for 30 seconds for 45 cycles.

Multi electrode array recordings

Cells were seeded into a 24-well multi electrode plate (24W300/30G-288 Multi Channel Systems) at a density of 100,000 cells per square centimeter over 12 electrodes per well. Cultures were fed twice a week with NBM +FABG3. Voltage recordings were taken immediately before or the day after feeding using the Multiwell-MEA system. The duration of each recording was two minutes. Active electrodes were determined as those that had more than three and less than 4000 spikes over the two-minute recording. Recordings were made on a Multiwell-MEA headstage (Multichannel Systems), in a 37 °C recording chamber. Data collection used Multiwell-Screen software (Multichannel Systems). Signals were high-pass filtered at 10 Hz and low-pass filtered at 3.5 kHz using inbuilt hardware filters. Each electrode voltage signal was further high-pass filtered at 300 Hz. Spikes were detected as rising or falling voltage deflections over 5x the standard deviation of the noise.

Patch-clamp electrophysiology

Coverslips containing SCPNs either cultured alone, or co-cultured with SMNs were used for whole-cell patch-clamp recordings 3-4 weeks after plating. Seven days after seeding, coverslips were removed from BrainPhys media, immediately placed in the recording chamber and constantly perfused with warmed artificial cerebrospinal fluid (aCSF) containing the following (in mM): 126 NaCl, 26 NaHCO3, 10 Glucose, 2.5 KCl, 1.25 NaHPO4, 2 CaCl2, 1 MgSO4 (bubbled with 95% CO2, 5% O2, pH. 7.4, 310 mOsm, 32ºC). Borosilicate glass capillaries were pulled to 3-5 MΩ tip resistance and filled with (in mM): 120 K-gluconate, 11 KCl, 10 HEPES, 1 MgCl2, 1 CaCl2, 1 EGTA, 0.1% Alexa Fluor 594 biocytin (pH. 7.4, 300 mOsm). Cells were visualized using a microscope equipped with DIC optics (Nikon Eclipse fn1). Electroresponsive properties of SCPNs were evaluated with gradually increasing current injections (-10 pA to 20 pA, 5 pA steps, 500 ms duration) while holding cells at -60 mV. Spontaneous excitatory postsynaptic currents (sEPSCs) were recorded in voltage-clamp at -60 mV. 20 µM DNQX disodium salt was added to the bath in a subset of recordings following a 5-minute baseline to block AMPA receptors. Recordings with > 20% change in the series resistance were discarded. Data was acquired using a MultiClamp 700B, digitized at 20 kHz with a Digidata 1550B, and analyzed using Clampfit 10.7 (Molecular Devices, LLC, San Jose, CA). sEPSCs were analyzed using NeuroExpress software v 21.1.13 [12].

Quantification of Synaptic Puncta

Synapse quantification was performed by measuring neuronal process length and counting puncta on FIJI using the NeuronJ plugin according to [13]. Neurite lengths identified as EGFP+ were traced, and synaptic puncta were counted by colocalization of GRIA1-4 and VGLUT1 signal along the neurite trace. For each pyramidal neuron the

number of synapses was divided by the length of its neurites in μm to give ten values of synapses per μm neurite. Then an unpaired t-test was used to compare these values from the two groups: subcerebral projection neurons (SCPN) in monoculture (n=10) versus SCPN in co-culture with spinal motor neurons (SCPN+SMN) (n=10), which yielded a p-value of 0.0021 (Fig. 6F). Antibodies used were guineapig anti-Synapsin1/2 Cat# 106004 from Synaptic Systems at 1:2K, chicken anti-GFP Cat# GFP-1010 from AVES at 1:2K, rabbit anti-VGLUT1 Cat# 135303 from Synaptic Systems at 1:1K, and mouse anti-GRIA1-4 Cat# MABN832 from Millipore at 1:1K dilution.

Supplemental Figures

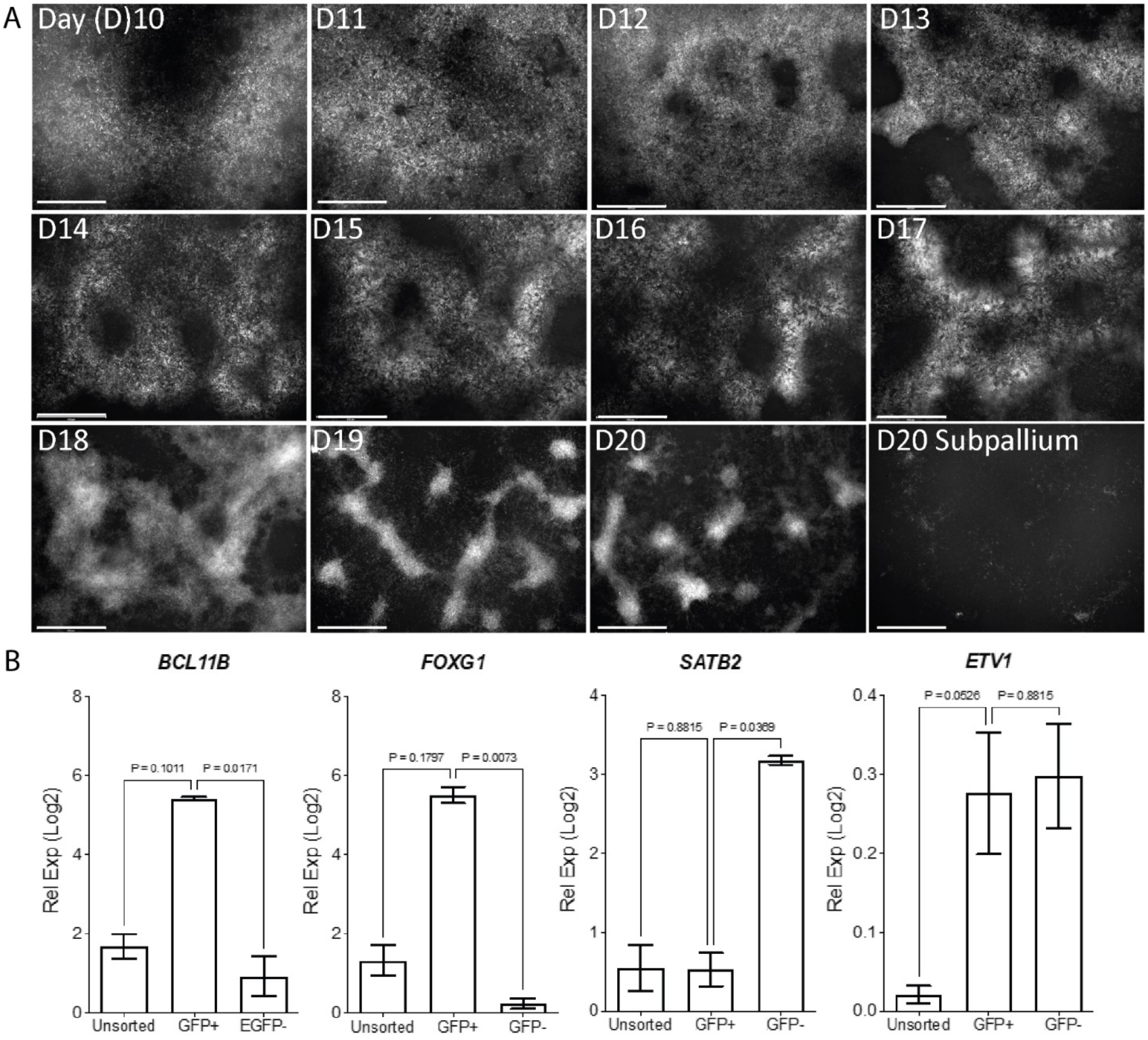

Figure S1. Integration of enhanced green fluorescent protein (GFP) reporter at FEZF2 locus enables isolation of populations that express CTIP2 and ETV1 while excluding SATB2, related to Figure 1. (A) GFP expression in live cultures undergoing directed cortical differentiation between day 10-20 at 4X. Scale bar is 650 µm. The final image is a parallel culture at day 20 undergoing ventral forebrain differentiation to enrich for sub-pallial domains [14]. (B) Relative gene expression of *BCL11B*, *FOXG1*, *SATB2*, and

ETV1 at differentiation day 21 in sorted populations normalized to *HPRT* (ΔCT) and then within samples to the lowest expressing sample (Log2) mean ± SEM of unsorted or sorted populations from three independent differentiations. Statistical analysis using one-way, non-parametric ANOVA with Kruskal-Wallis test and Uncorrected Dunn's test.

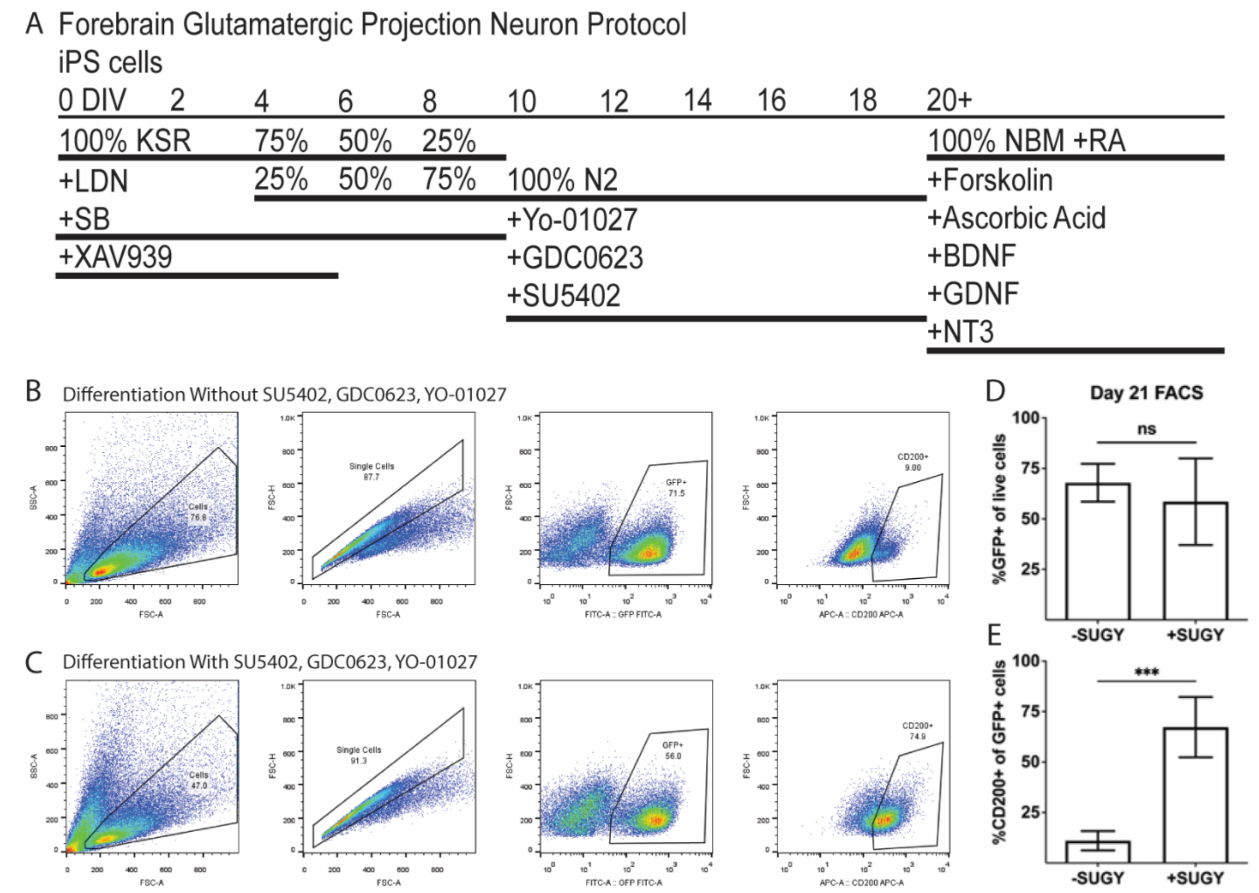

Figure S2. Three small molecule inhibitors **enhance expression of** ***CD200 within the GFP+ population*** (A) Diagram outlining the protocol used to differentiate hiPSC, introducing knockout serum replacement medium (KSR) from day 0 to day 4; introducing 25% N2 medium increase on day 4, 50% on day 6, 75% on day 8, and100% N2 medium on day 10. and introducing neurobasal-A medium (NBM) from day 20 onward. (B-C) Gating strategy used in fluorescence activated cell sorting (FACS) for viable cells by forward scatter area (FSC-A) and side scatter area (SSC-A), gating for single cells, GFP+ by Fluorescein isothiocyanate area (FITC-A) and forward scatter height (FSC-H), CD200+ by Allophycocyanin area (APC-A) and FSC-H. The same gating strategy was used for differentiations without the three inhibitors (B) or with the SU5402, GDC0623, and YO-01027 (SUGY) treatment (C). We chose these specific small molecules due to

their high efficacy, thermostability, and low toxicity. (D-E) Bar graph quantification of the FACS percentage positive of GFP (D) and CD200 (E) events displayed as mean ± SD of $n \geq 7$ independent differentiations for each protocol (i.e. -/+SUGY) Statistical significance was assessed using a Mann–Whitney U test, ns $p = 0.2866$, *** $p = 0.0002$.

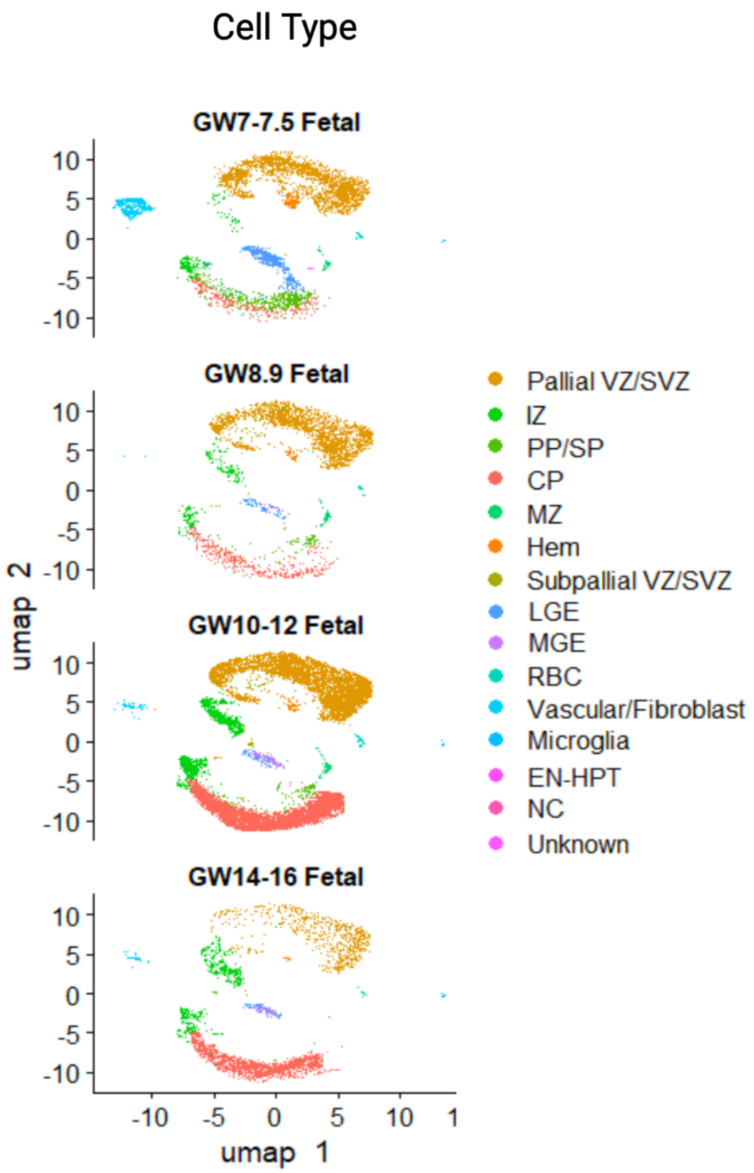

Figure S3. Fetal data subset from the integrated datasets (Fig. 2) split by sample group corresponding to fetal age in gestational weeks (GW) with clusters color-coded to associate with developmental regions of the forebrain.

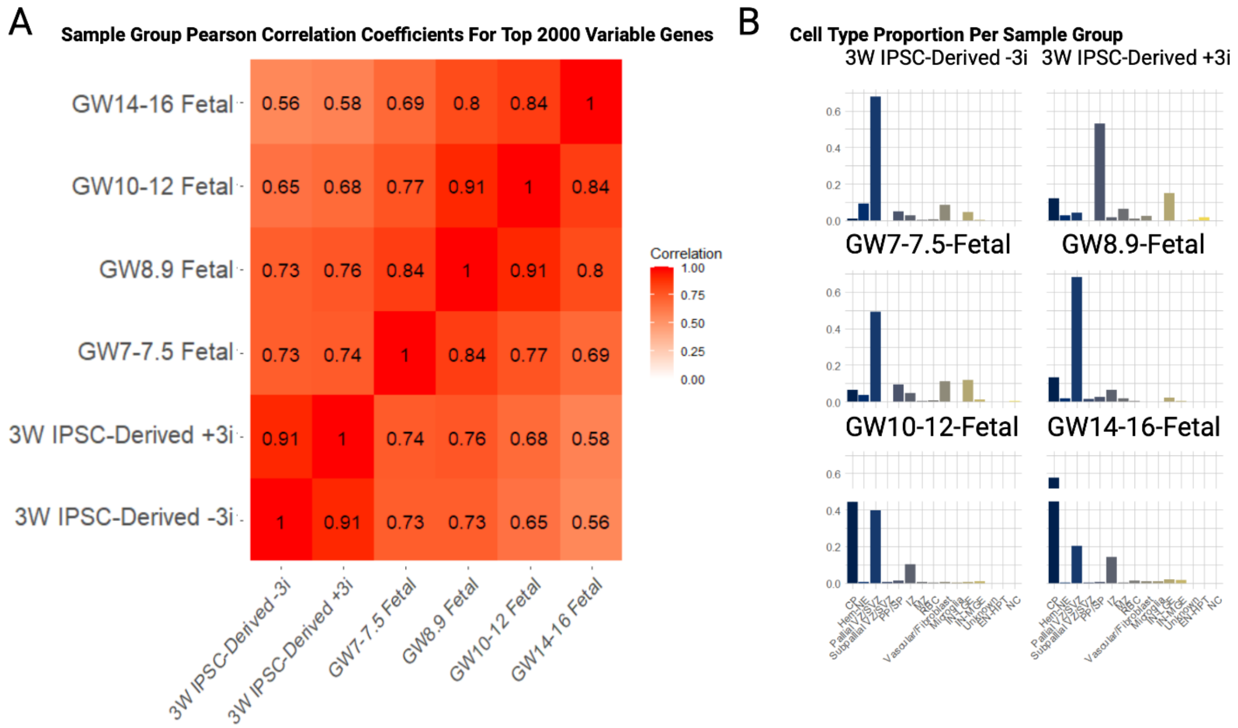

	3W IPSC-Derived -3i	3W IPSC-Derived +3i	GW7-7.5 Fetal	GW8.9 Fetal	GW10-12 Fetal	GW14-16 Fetal
GW14-16 Fetal	0.56	0.58	0.69	0.8	0.84	1
GW10-12 Fetal	0.65	0.68	0.77	0.91	1	0.84
GW8.9 Fetal	0.73	0.76	0.84	1	0.91	0.8
GW7-7.5 Fetal	0.73	0.74	1	0.84	0.77	0.69
3W IPSC-Derived +3i	0.91	1	0.74	0.76	0.68	0.58
3W IPSC-Derived -3i	1	0.91	0.73	0.73	0.65	0.56

Figure S4. Correlation of Gene Expression and Cell Type Proportion of 3 Week hiPSC-Derived Cells with Different Fetal Stages (A) Comparisons of the Pearson correlation coefficients between the normalized expression levels of the 2000 most variable genes within each group pair. (B). Proportions of each integrated cell type cluster within each sample group.

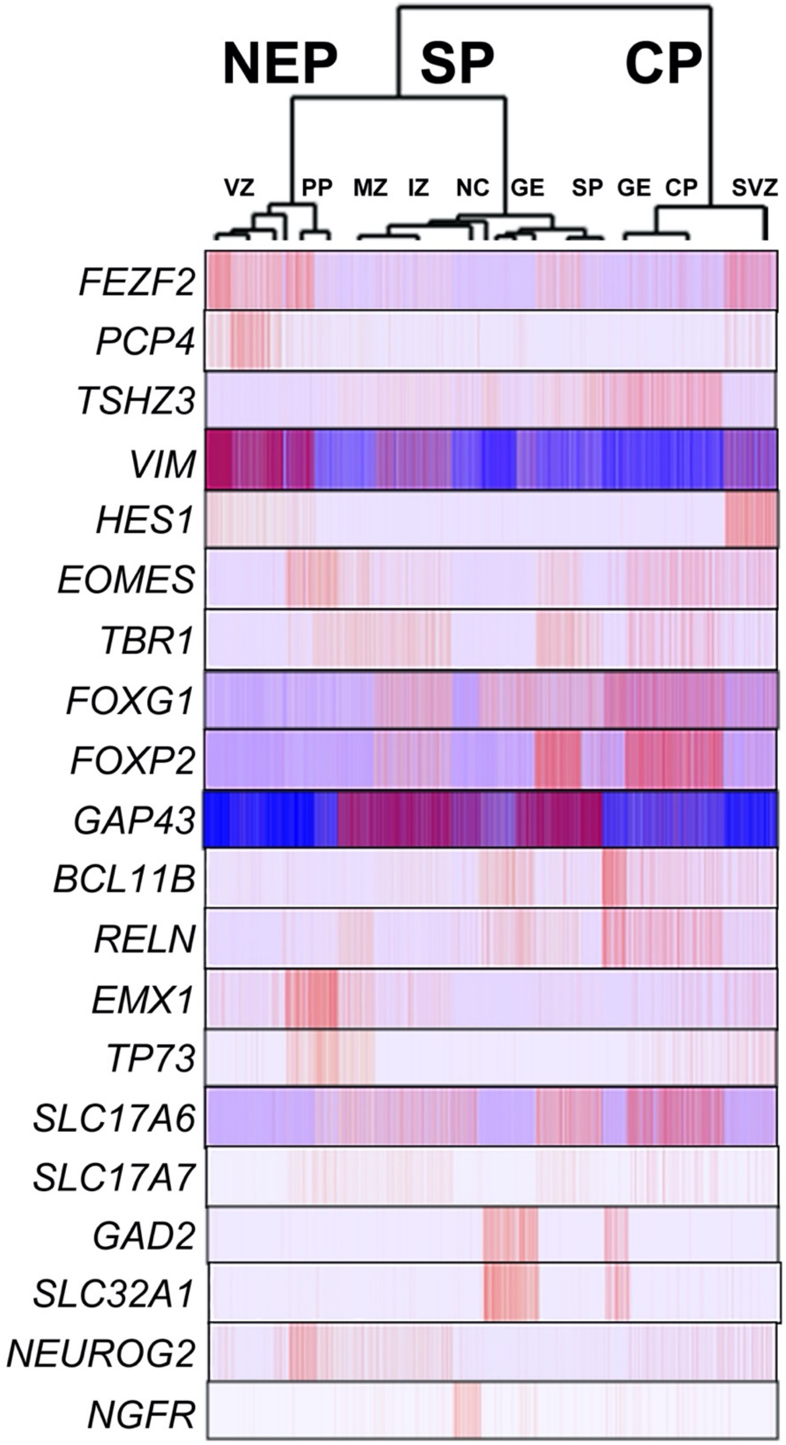

Figure S5. Single cell gene expression signatures correlate with fetal developmental regions that coincide with hierarchical clustering branches.

PAGODA analyses showing specific gene expression signatures that correlate with populations separated by hierarchical clustering branches.

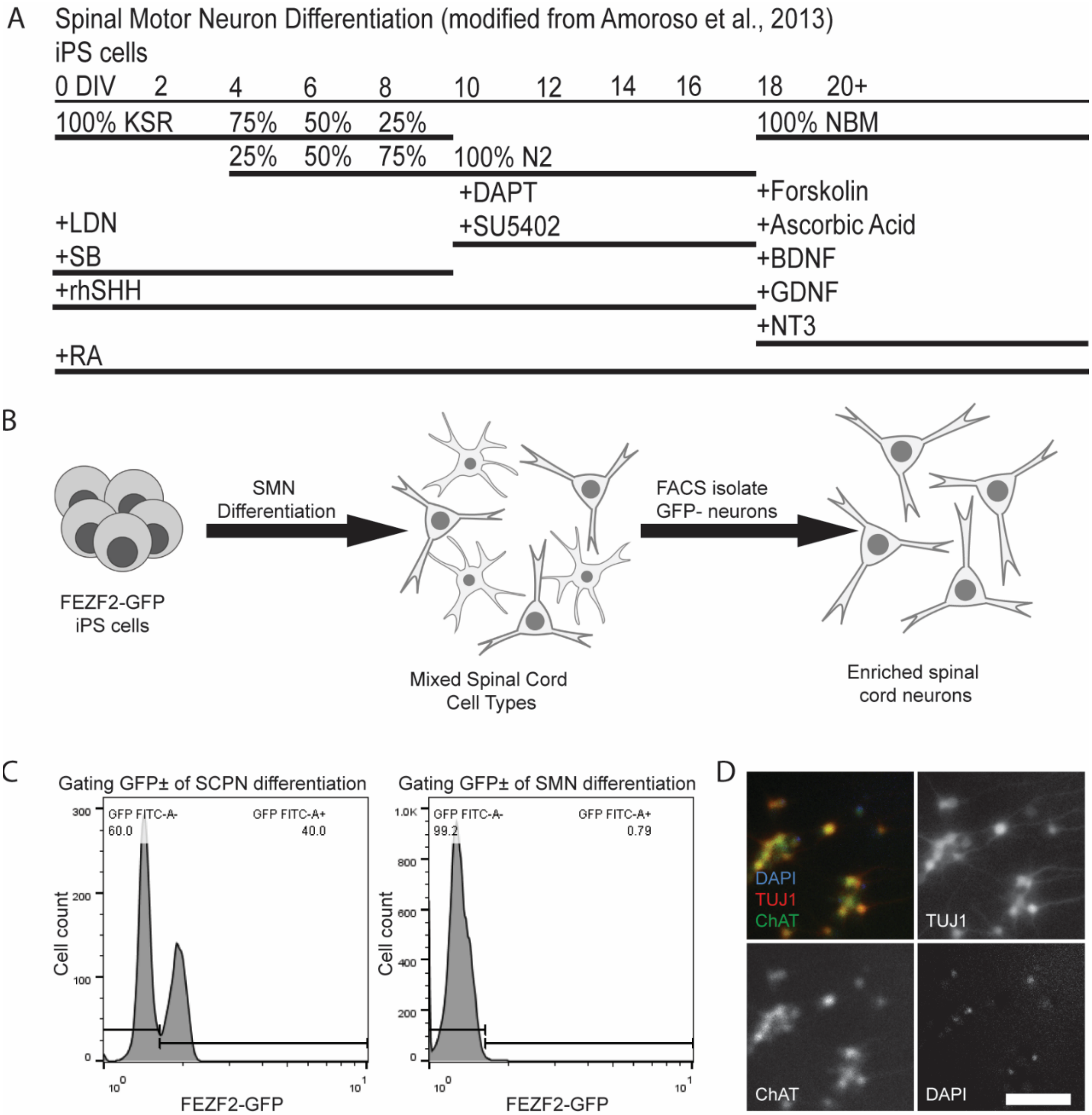

Figure S6. Spinal motor neuron (SMN) differentiation and enrichment of FEZF2-GFP negative SMN. (A) SMN differentiation protocol modified from [15]. (B) Schematic depicting the workflow to enrich spinal cord neurons from FEZF2-GFP iPSC line for use in co-culture experiments with cortical FEZF2-GFP positive SCPNs generated from the same iPSC line. (C) FACS gates of FEZF2-GFP positive or negative cells from SCPN or SMN differentiations, respectively. (D) Representative 20X objective image of FACS-enriched

spinal cord neurons from SMN differentiation showing DAPI+ (blue), neuron-specific beta-III Tubulin (TUJ1)+ (red), and the cholinergic neuron marker choline acetyltransferase (ChAT)+ (green) cells, scale bar is 50 μm.

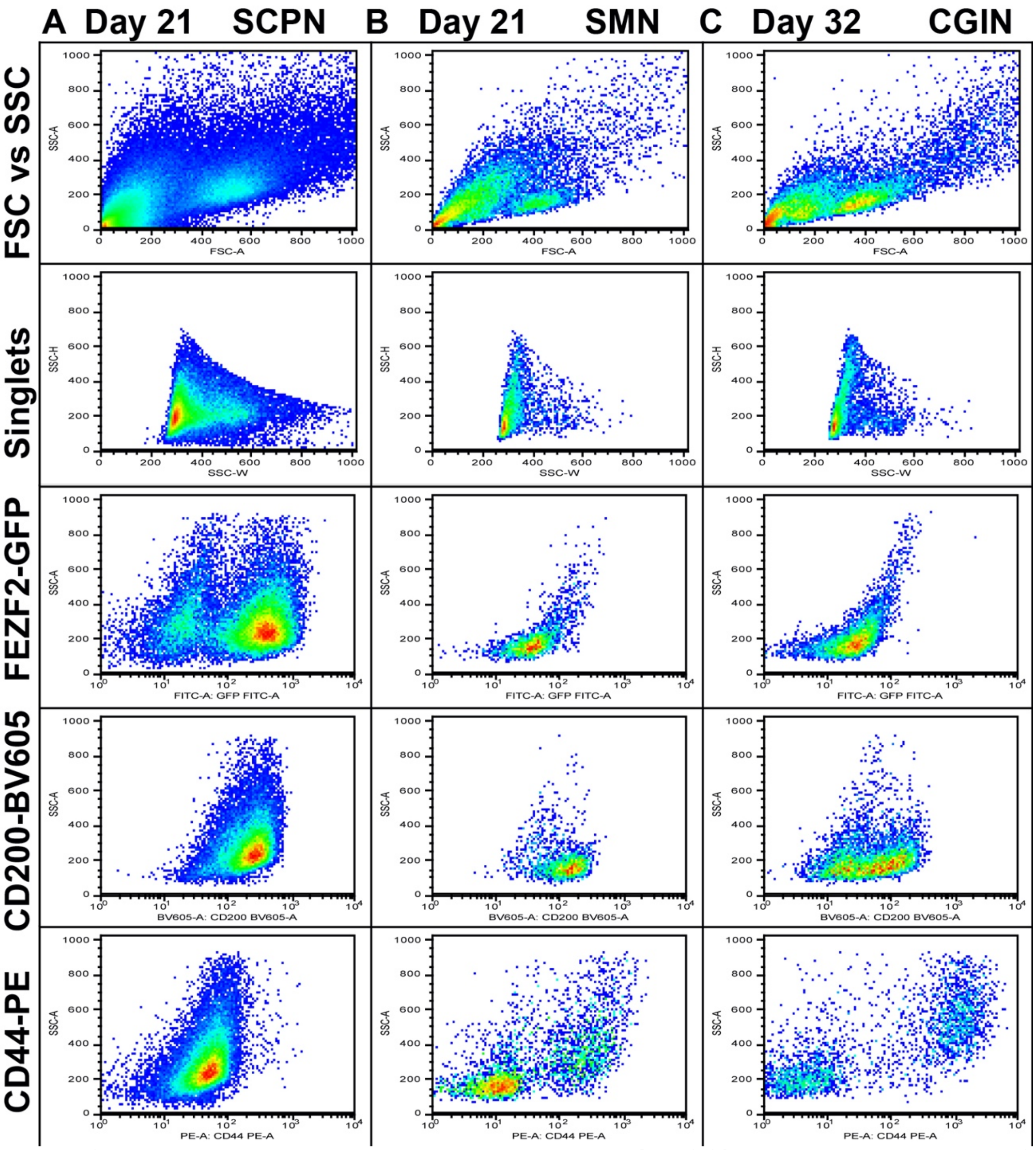

Figure S7. Fluorescence activated cell sorting (FACS)-based enrichment of spinal motor neurons (SMN) and cortical GABAergic interneurons (CGIN) from FEZF2-GFP negative cells. (A) Sub-cerebral projection neurons (SCPN) after 21 days of differentiation enrich for FEZF2-GFP, CD200, and sparse CD44+ labeling. (B) SMN differentiated for 21 days [15] do not express FEZF2-GFP and enrich for CD200 and CD44. (C) CGIN differentiated for 32 days [15] do not express FEZF2-GFP and enrich for CD200

and CD44. SMN and CGIN were sorted for GFP-/CD200+/CD44- cells and replated for co-culture experiments in figure 6 and figure 7.

Table S1. List of antibodies used.

Host species	Antigen	Cat#	Manufacturer	RRID	Concen-tration
AF488-Donkey	anti-Chicken	A78948	Invitrogen	AB_2921070	1 µg/mL
AF647 Donkey	anti-Guinea pig	SA000059	Invitrogen	-	1 µg/mL
AF405 Donkey	anti-Guinea pig	SA000005	Invitrogen	-	1 µg/mL
AF555 Donkey	anti-Guinea pig	SA000038	Invitrogen	-	1 µg/mL
AF555 Donkey	anti-Mouse	A-31570	Invitrogen	AB_2536180	1 µg/mL
AF647 Donkey	anti-Mouse	A-31571	Invitrogen	AB_162542	1 µg/mL
AF405 Donkey	anti-Rabbit	SA000006	Invitrogen	-	1 µg/mL
AF488 Donkey	anti-Rabbit	A-21206	Invitrogen	AB_2535792	1 µg/mL
AF555 Donkey	anti-Rabbit	A-31572	Invitrogen	AB_162543	1 µg/mL
AF647 Donkey	anti-Rabbit	A-31573	Invitrogen	AB_2536183	1 µg/mL
AF555 Donkey	anti-Rat	A78945	Invitrogen	AB_2910652	1 µg/mL
AF647 Donkey	anti-Rat	A78947	Invitrogen	AB_2910635	1 µg/mL
Mouse	Beta-3 Tubulin (TUJ1)	MMS-435P	BioLegend	AB_2313773	1:2000
Mouse	CD200-BV605	329218	BioLegend	AB_2563370	5 µl/10^6 cells
Mouse	CD200-APC	FAB27241A	R&D Systems/ Bio-Techne		10 µl/10^6 cells
Rat	CD44-PE	103024	BioLegend	AB_312958	2 µl/10^6 cells
Goat	ChAT	AB144P	Sigma	-	1:200
Rat	CTIP2 (BCL11B)	AB18465	Abcam	-	1:500
Chicken	EGFP	GFP-1010	Aves Labs	AB_2307313	1:2000
Rabbit	EGFP	AB6556	ABCAM	-	1:2000
Mouse	GRIA1-4	MABN832	Millipore	-	1:1000
Guinea pig	NEUN	266 004	Synaptic Systems	-	1:500
Mouse	NEUN	MAB377	Sigma	-	-

Mouse	SATB2	AB51502	Abcam	-	0.1 - 1 mg/mL
Guinea pig	Synapsin1/2	106004	Synaptic Systems	-	1:2000
Rabbit	TBR1	AB31940	ABCAM	-	0.8 - 1 mg/mL
Rabbit	VGLUT1 (SLC17A7)	135303	Synaptic Systems	-	1:1000

Table S2. Intrinsic membrane properties of iPSC-derived FEZF2-GFP+ subcerebral projection neurons (SCPN) cultured alone or in the presence of spinal motor neurons (SMN).

	Vrest (mV)	Spike Threshold (mV)	Spike Amplitude (mV)	AHP (mV)	Tau (ms)	Half-max (ms)	Rin (MΩ)	Rheobase
SCPN	-47.1538 ± 11.613	-35.6422 ± 7.842	74.0706 ± 12.199	-61.6362 ± 4.8165	90.4292 ± 43.744	1.7881 ± 0.6345	1178.4322 ± 640.54	14.4231 ± 12.754
SCPN +SMN	-52.6667 ± 9.004	-35.2858 ± 5.223	82.0570 ± 11.761	-60.2284 ± 5.749	70.9965 ± 31.195	1.3467 ± 0.3457	982.5283 ± 533.87	14.3590 ± 10.399
p-value	0.0176	0.4132	0.0052	0.1638	0.0202	0.0003	0.0930	0.4912

Supplemental References

1. Kiskinis E, Sandoe J, Williams LA, et al. Pathways disrupted in human ALS motor neurons identified through genetic correction of mutant SOD1. Cell stem cell 2014;14(6):781-795
2. Boulting GL, Kiskinis E, Croft GF, et al. A functionally characterized test set of human induced pluripotent stem cells. Nature biotechnology 2011;29(3):279
3. Sanjana NE, Cong L, Zhou Y, et al. A transcription activator-like effector toolbox for genome engineering. Nature protocols 2012;7(1):171
4. Hermann BP, Cheng K, Singh A, et al. The mammalian spermatogenesis single-cell transcriptome, from spermatogonial stem cells to spermatids. Cell reports 2018;25(6):1650-1667. e8
5. Butler A, Hoffman P, Smibert P, et al. Integrating single-cell transcriptomic data across different conditions, technologies, and species. Nature biotechnology 2018;36(5):411-420
6. Hao Y, Hao S, Andersen-Nissen E, et al. Integrated analysis of multimodal single-cell data. Cell 2021;184(13):3573-3587. e29
7. La Manno G, Soldatov R, Zeisel A, et al. RNA velocity of single cells. Nature 2018;560(7719):494-498
8. Svensson V, Pachter L. RNA velocity: molecular kinetics from single-cell RNA-Seq. Molecular cell 2018;72(1):7-9
9. Barkas N, Petukhov V, Nikolaeva D, et al. Joint analysis of heterogeneous single-cell RNA-seq dataset collections. Nature methods 2019;16(8):695-698
10. Chen EY, Tan CM, Kou Y, et al. Enrichr: interactive and collaborative HTML5 gene list enrichment analysis tool. BMC bioinformatics 2013;14(1-14
11. Kuleshov MV, Jones MR, Rouillard AD, et al. Enrichr: a comprehensive gene set enrichment analysis web server 2016 update. Nucleic acids research 2016;44(W1):W90-W97
12. Szücs A. NeuroExpress program for analyzing patch-clamp data. ResearchGate: 2022.
13. Meijering E, Jacob M, Sarria JC, et al. Design and validation of a tool for neurite tracing and analysis in fluorescence microscopy images. Cytometry Part A: the journal of the International Society for Analytical Cytology 2004;58(2):167-176
14. Maroof AM, Keros S, Tyson JA, et al. Directed differentiation and functional maturation of cortical interneurons from human embryonic stem cells. Cell stem cell 2013;12(5):559-572
15. Amoroso MW, Croft GF, Williams DJ, et al. Accelerated High-Yield Generation of Limb-Innervating Motor Neurons from Human Stem Cells. The Journal of Neuroscience 2013;33(2):574-586, doi:10.1523/jneurosci.0906-12.2013
